# Unveiling the strong interaction among hadrons at the LHC

**DOI:** 10.1038/s41586-020-3001-6

**Published:** 2020-12-09

**Authors:** ALICE Collaboration, S. Acharya, D. Adamová, A. Adler, J. Adolfsson, M. M. Aggarwal, G. Aglieri Rinella, M. Agnello, N. Agrawal, Z. Ahammed, S. Ahmad, S. U. Ahn, Z. Akbar, A. Akindinov, M. Al-Turany, S. N. Alam, D. S. D. Albuquerque, D. Aleksandrov, B. Alessandro, H. M. Alfanda, R. Alfaro Molina, B. Ali, Y. Ali, A. Alici, N. Alizadehvandchali, A. Alkin, J. Alme, T. Alt, L. Altenkamper, I. Altsybeev, M. N. Anaam, C. Andrei, D. Andreou, A. Andronic, M. Angeletti, V. Anguelov, C. Anson, T. Antičić, F. Antinori, P. Antonioli, N. Apadula, L. Aphecetche, H. Appelshäuser, S. Arcelli, R. Arnaldi, M. Arratia, I. C. Arsene, M. Arslandok, A. Augustinus, R. Averbeck, S. Aziz, M. D. Azmi, A. Badalà, Y. W. Baek, S. Bagnasco, X. Bai, R. Bailhache, R. Bala, A. Balbino, A. Baldisseri, M. Ball, S. Balouza, D. Banerjee, R. Barbera, L. Barioglio, G. G. Barnaföldi, L. S. Barnby, V. Barret, P. Bartalini, C. Bartels, K. Barth, E. Bartsch, F. Baruffaldi, N. Bastid, S. Basu, G. Batigne, B. Batyunya, D. Bauri, J. L. Bazo Alba, I. G. Bearden, C. Beattie, C. Bedda, N. K. Behera, I. Belikov, A. D. C. Bell Hechavarria, F. Bellini, R. Bellwied, V. Belyaev, G. Bencedi, S. Beole, A. Bercuci, Y. Berdnikov, D. Berenyi, R. A. Bertens, D. Berzano, M. G. Besoiu, L. Betev, A. Bhasin, I. R. Bhat, M. A. Bhat, H. Bhatt, B. Bhattacharjee, A. Bianchi, L. Bianchi, N. Bianchi, J. Bielčík, J. Bielčíková, A. Bilandzic, G. Biro, R. Biswas, S. Biswas, J. T. Blair, D. Blau, C. Blume, G. Boca, F. Bock, A. Bogdanov, S. Boi, J. Bok, L. Boldizsár, A. Bolozdynya, M. Bombara, G. Bonomi, H. Borel, A. Borissov, H. Bossi, E. Botta, L. Bratrud, P. Braun-Munzinger, M. Bregant, M. Broz, E. Bruna, G. E. Bruno, M. D. Buckland, D. Budnikov, H. Buesching, S. Bufalino, O. Bugnon, P. Buhler, P. Buncic, Z. Buthelezi, J. B. Butt, S. A. Bysiak, D. Caffarri, A. Caliva, E. Calvo Villar, J. M. M. Camacho, R. S. Camacho, P. Camerini, F. D. M. Canedo, A. A. Capon, F. Carnesecchi, R. Caron, J. Castillo Castellanos, A. J. Castro, E. A. R. Casula, F. Catalano, C. Ceballos Sanchez, P. Chakraborty, S. Chandra, W. Chang, S. Chapeland, M. Chartier, S. Chattopadhyay, S. Chattopadhyay, A. Chauvin, C. Cheshkov, B. Cheynis, V. Chibante Barroso, D. D. Chinellato, S. Cho, P. Chochula, T. Chowdhury, P. Christakoglou, C. H. Christensen, P. Christiansen, T. Chujo, C. Cicalo, L. Cifarelli, L. D. Cilladi, F. Cindolo, M. R. Ciupek, G. Clai, J. Cleymans, F. Colamaria, D. Colella, A. Collu, M. Colocci, M. Concas, G. Conesa Balbastre, Z. Conesa del Valle, G. Contin, J. G. Contreras, T. M. Cormier, Y. Corrales Morales, P. Cortese, M. R. Cosentino, F. Costa, S. Costanza, P. Crochet, E. Cuautle, P. Cui, L. Cunqueiro, D. Dabrowski, T. Dahms, A. Dainese, F. P. A. Damas, M. C. Danisch, A. Danu, D. Das, I. Das, P. Das, P. Das, S. Das, A. Dash, S. Dash, S. De, A. De Caro, G. de Cataldo, J. de Cuveland, A. De Falco, D. De Gruttola, N. De Marco, S. De Pasquale, S. Deb, H. F. Degenhardt, K. R. Deja, A. Deloff, S. Delsanto, W. Deng, P. Dhankher, D. Di Bari, A. Di Mauro, R. A. Diaz, T. Dietel, P. Dillenseger, Y. Ding, R. Divià, D. U. Dixit, Ø. Djuvsland, U. Dmitrieva, A. Dobrin, B. Dönigus, O. Dordic, A. K. Dubey, A. Dubla, S. Dudi, M. Dukhishyam, P. Dupieux, R. J. Ehlers, V. N. Eikeland, D. Elia, B. Erazmus, F. Erhardt, A. Erokhin, M. R. Ersdal, B. Espagnon, G. Eulisse, D. Evans, S. Evdokimov, L. Fabbietti, M. Faggin, J. Faivre, F. Fan, A. Fantoni, M. Fasel, P. Fecchio, A. Feliciello, G. Feofilov, A. Fernández Téllez, A. Ferrero, A. Ferretti, A. Festanti, V. J. G. Feuillard, J. Figiel, S. Filchagin, D. Finogeev, F. M. Fionda, G. Fiorenza, F. Flor, A. N. Flores, S. Foertsch, P. Foka, S. Fokin, E. Fragiacomo, U. Frankenfeld, U. Fuchs, C. Furget, A. Furs, M. Fusco Girard, J. J. Gaardhøje, M. Gagliardi, A. M. Gago, A. Gal, C. D. Galvan, P. Ganoti, C. Garabatos, J. R. A. Garcia, E. Garcia-Solis, K. Garg, C. Gargiulo, A. Garibli, K. Garner, P. Gasik, E. F. Gauger, M. B. Gay Ducati, M. Germain, J. Ghosh, P. Ghosh, S. K. Ghosh, M. Giacalone, P. Gianotti, P. Giubellino, P. Giubilato, A. M. C. Glaenzer, P. Glässel, A. Gomez Ramirez, V. Gonzalez, L. H. González-Trueba, S. Gorbunov, L. Görlich, A. Goswami, S. Gotovac, V. Grabski, L. K. Graczykowski, K. L. Graham, L. Greiner, A. Grelli, C. Grigoras, V. Grigoriev, A. Grigoryan, S. Grigoryan, O. S. Groettvik, F. Grosa, J. F. Grosse-Oetringhaus, R. Grosso, R. Guernane, M. Guittiere, K. Gulbrandsen, T. Gunji, A. Gupta, R. Gupta, I. B. Guzman, R. Haake, M. K. Habib, C. Hadjidakis, H. Hamagaki, G. Hamar, M. Hamid, R. Hannigan, M. R. Haque, A. Harlenderova, J. W. Harris, A. Harton, J. A. Hasenbichler, H. Hassan, Q. U. Hassan, D. Hatzifotiadou, P. Hauer, L. B. Havener, S. Hayashi, S. T. Heckel, E. Hellbär, H. Helstrup, A. Herghelegiu, T. Herman, E. G. Hernandez, G. Herrera Corral, F. Herrmann, K. F. Hetland, H. Hillemanns, C. Hills, B. Hippolyte, B. Hohlweger, J. Honermann, D. Horak, A. Hornung, S. Hornung, R. Hosokawa, P. Hristov, C. Huang, C. Hughes, P. Huhn, T. J. Humanic, H. Hushnud, L. A. Husova, N. Hussain, S. A. Hussain, D. Hutter, J. P. Iddon, R. Ilkaev, H. Ilyas, M. Inaba, G. M. Innocenti, M. Ippolitov, A. Isakov, M. S. Islam, M. Ivanov, V. Ivanov, V. Izucheev, B. Jacak, N. Jacazio, P. M. Jacobs, S. Jadlovska, J. Jadlovsky, S. Jaelani, C. Jahnke, M. J. Jakubowska, M. A. Janik, T. Janson, M. Jercic, O. Jevons, M. Jin, F. Jonas, P. G. Jones, J. Jung, M. Jung, A. Jusko, P. Kalinak, A. Kalweit, V. Kaplin, S. Kar, A. Karasu Uysal, D. Karatovic, O. Karavichev, T. Karavicheva, P. Karczmarczyk, E. Karpechev, A. Kazantsev, U. Kebschull, R. Keidel, M. Keil, B. Ketzer, Z. Khabanova, A. M. Khan, S. Khan, A. Khanzadeev, Y. Kharlov, A. Khatun, A. Khuntia, B. Kileng, B. Kim, B. Kim, D. Kim, D. J. Kim, E. J. Kim, H. Kim, J. Kim, J. S. Kim, J. Kim, J. Kim, J. Kim, M. Kim, S. Kim, T. Kim, T. Kim, S. Kirsch, I. Kisel, S. Kiselev, A. Kisiel, J. L. Klay, C. Klein, J. Klein, S. Klein, C. Klein-Bösing, M. Kleiner, A. Kluge, M. L. Knichel, A. G. Knospe, C. Kobdaj, M. K. Köhler, T. Kollegger, A. Kondratyev, N. Kondratyeva, E. Kondratyuk, J. Konig, S. A. Konigstorfer, P. J. Konopka, G. Kornakov, L. Koska, O. Kovalenko, V. Kovalenko, M. Kowalski, I. Králik, A. Kravčáková, L. Kreis, M. Krivda, F. Krizek, K. Krizkova Gajdosova, M. Krüger, E. Kryshen, M. Krzewicki, A. M. Kubera, V. Kučera, C. Kuhn, P. G. Kuijer, L. Kumar, S. Kundu, P. Kurashvili, A. Kurepin, A. B. Kurepin, A. Kuryakin, S. Kushpil, J. Kvapil, M. J. Kweon, J. Y. Kwon, Y. Kwon, S. L. La Pointe, P. La Rocca, Y. S. Lai, M. Lamanna, R. Langoy, K. Lapidus, A. Lardeux, P. Larionov, E. Laudi, R. Lavicka, T. Lazareva, R. Lea, L. Leardini, J. Lee, S. Lee, S. Lehner, J. Lehrbach, R. C. Lemmon, I. León Monzón, E. D. Lesser, M. Lettrich, P. Lévai, X. Li, X. L. Li, J. Lien, R. Lietava, B. Lim, V. Lindenstruth, A. Lindner, C. Lippmann, M. A. Lisa, A. Liu, J. Liu, S. Liu, W. J. Llope, I. M. Lofnes, V. Loginov, C. Loizides, P. Loncar, J. A. Lopez, X. Lopez, E. López Torres, J. R. Luhder, M. Lunardon, G. Luparello, Y. G. Ma, A. Maevskaya, M. Mager, S. M. Mahmood, T. Mahmoud, A. Maire, R. D. Majka, M. Malaev, Q. W. Malik, L. Malinina, D. Mal’Kevich, P. Malzacher, G. Mandaglio, V. Manko, F. Manso, V. Manzari, Y. Mao, M. Marchisone, J. Mareš, G. V. Margagliotti, A. Margotti, A. Marín, C. Markert, M. Marquard, C. D. Martin, N. A. Martin, P. Martinengo, J. L. Martinez, M. I. Martínez, G. Martínez García, S. Masciocchi, M. Masera, A. Masoni, L. Massacrier, E. Masson, A. Mastroserio, A. M. Mathis, O. Matonoha, P. F. T. Matuoka, A. Matyja, C. Mayer, F. Mazzaschi, M. Mazzilli, M. A. Mazzoni, A. F. Mechler, F. Meddi, Y. Melikyan, A. Menchaca-Rocha, C. Mengke, E. Meninno, A. S. Menon, M. Meres, S. Mhlanga, Y. Miake, L. Micheletti, L. C. Migliorin, D. L. Mihaylov, K. Mikhaylov, A. N. Mishra, D. Miśkowiec, A. Modak, N. Mohammadi, A. P. Mohanty, B. Mohanty, M. Mohisin Khan, Z. Moravcova, C. Mordasini, D. A. Moreira De Godoy, L. A. P. Moreno, I. Morozov, A. Morsch, T. Mrnjavac, V. Muccifora, E. Mudnic, D. Mühlheim, S. Muhuri, J. D. Mulligan, A. Mulliri, M. G. Munhoz, R. H. Munzer, H. Murakami, S. Murray, L. Musa, J. Musinsky, C. J. Myers, J. W. Myrcha, B. Naik, R. Nair, B. K. Nandi, R. Nania, E. Nappi, M. U. Naru, A. F. Nassirpour, C. Nattrass, R. Nayak, T. K. Nayak, S. Nazarenko, A. Neagu, R. A. Negrao De Oliveira, L. Nellen, S. V. Nesbo, G. Neskovic, D. Nesterov, L. T. Neumann, B. S. Nielsen, S. Nikolaev, S. Nikulin, V. Nikulin, F. Noferini, P. Nomokonov, J. Norman, N. Novitzky, P. Nowakowski, A. Nyanin, J. Nystrand, M. Ogino, A. Ohlson, J. Oleniacz, A. C. Oliveira Da Silva, M. H. Oliver, C. Oppedisano, A. Ortiz Velasquez, A. Oskarsson, J. Otwinowski, K. Oyama, Y. Pachmayer, V. Pacik, S. Padhan, D. Pagano, G. Paić, J. Pan, S. Panebianco, P. Pareek, J. Park, J. E. Parkkila, S. Parmar, S. P. Pathak, B. Paul, J. Pazzini, H. Pei, T. Peitzmann, X. Peng, L. G. Pereira, H. Pereira Da Costa, D. Peresunko, G. M. Perez, S. Perrin, Y. Pestov, V. Petráček, M. Petrovici, R. P. Pezzi, S. Piano, M. Pikna, P. Pillot, O. Pinazza, L. Pinsky, C. Pinto, S. Pisano, D. Pistone, M. Płoskoń, M. Planinic, F. Pliquett, M. G. Poghosyan, B. Polichtchouk, N. Poljak, A. Pop, S. Porteboeuf-Houssais, V. Pozdniakov, S. K. Prasad, R. Preghenella, F. Prino, C. A. Pruneau, I. Pshenichnov, M. Puccio, J. Putschke, S. Qiu, L. Quaglia, R. E. Quishpe, S. Ragoni, S. Raha, S. Rajput, J. Rak, A. Rakotozafindrabe, L. Ramello, F. Rami, S. A. R. Ramirez, R. Raniwala, S. Raniwala, S. S. Räsänen, R. Rath, V. Ratza, I. Ravasenga, K. F. Read, A. R. Redelbach, K. Redlich, A. Rehman, P. Reichelt, F. Reidt, X. Ren, R. Renfordt, Z. Rescakova, K. Reygers, A. Riabov, V. Riabov, T. Richert, M. Richter, P. Riedler, W. Riegler, F. Riggi, C. Ristea, S. P. Rode, M. Rodríguez Cahuantzi, K. Røed, R. Rogalev, E. Rogochaya, D. Rohr, D. Röhrich, P. F. Rojas, P. S. Rokita, F. Ronchetti, A. Rosano, E. D. Rosas, K. Roslon, A. Rossi, A. Rotondi, A. Roy, P. Roy, O. V. Rueda, R. Rui, B. Rumyantsev, A. Rustamov, E. Ryabinkin, Y. Ryabov, A. Rybicki, H. Rytkonen, O. A. M. Saarimaki, R. Sadek, S. Sadhu, S. Sadovsky, K. Šafařík, S. K. Saha, B. Sahoo, P. Sahoo, R. Sahoo, S. Sahoo, P. K. Sahu, J. Saini, S. Sakai, S. Sambyal, V. Samsonov, D. Sarkar, N. Sarkar, P. Sarma, V. M. Sarti, M. H. P. Sas, E. Scapparone, J. Schambach, H. S. Scheid, C. Schiaua, R. Schicker, A. Schmah, C. Schmidt, H. R. Schmidt, M. O. Schmidt, M. Schmidt, N. V. Schmidt, A. R. Schmier, J. Schukraft, Y. Schutz, K. Schwarz, K. Schweda, G. Scioli, E. Scomparin, J. E. Seger, Y. Sekiguchi, D. Sekihata, I. Selyuzhenkov, S. Senyukov, D. Serebryakov, A. Sevcenco, A. Shabanov, A. Shabetai, R. Shahoyan, W. Shaikh, A. Shangaraev, A. Sharma, A. Sharma, H. Sharma, M. Sharma, N. Sharma, S. Sharma, O. Sheibani, K. Shigaki, M. Shimomura, S. Shirinkin, Q. Shou, Y. Sibiriak, S. Siddhanta, T. Siemiarczuk, D. Silvermyr, G. Simatovic, G. Simonetti, B. Singh, R. Singh, R. Singh, R. Singh, V. K. Singh, V. Singhal, T. Sinha, B. Sitar, M. Sitta, T. B. Skaali, M. Slupecki, N. Smirnov, R. J. M. Snellings, C. Soncco, J. Song, A. Songmoolnak, F. Soramel, S. Sorensen, I. Sputowska, J. Stachel, I. Stan, P. J. Steffanic, E. Stenlund, S. F. Stiefelmaier, D. Stocco, M. M. Storetvedt, L. D. Stritto, A. A. P. Suaide, T. Sugitate, C. Suire, M. Suleymanov, M. Suljic, R. Sultanov, M. Šumbera, V. Sumberia, S. Sumowidagdo, S. Swain, A. Szabo, I. Szarka, U. Tabassam, S. F. Taghavi, G. Taillepied, J. Takahashi, G. J. Tambave, S. Tang, M. Tarhini, M. G. Tarzila, A. Tauro, G. Tejeda Muñoz, A. Telesca, L. Terlizzi, C. Terrevoli, D. Thakur, S. Thakur, D. Thomas, F. Thoresen, R. Tieulent, A. Tikhonov, A. R. Timmins, A. Toia, N. Topilskaya, M. Toppi, F. Torales-Acosta, S. R. Torres, A. Trifiró, S. Tripathy, T. Tripathy, S. Trogolo, G. Trombetta, L. Tropp, V. Trubnikov, W. H. Trzaska, T. P. Trzcinski, B. A. Trzeciak, A. Tumkin, R. Turrisi, T. S. Tveter, K. Ullaland, E. N. Umaka, A. Uras, G. L. Usai, M. Vala, N. Valle, S. Vallero, N. van der Kolk, L. V. R. van Doremalen, M. van Leeuwen, P. Vande Vyvre, D. Varga, Z. Varga, M. Varga-Kofarago, A. Vargas, M. Vasileiou, A. Vasiliev, O. Vázquez Doce, V. Vechernin, E. Vercellin, S. Vergara Limón, L. Vermunt, R. Vernet, R. Vértesi, L. Vickovic, Z. Vilakazi, O. Villalobos Baillie, G. Vino, A. Vinogradov, T. Virgili, V. Vislavicius, A. Vodopyanov, B. Volkel, M. A. Völkl, K. Voloshin, S. A. Voloshin, G. Volpe, B. von Haller, I. Vorobyev, D. Voscek, J. Vrláková, B. Wagner, M. Weber, S. G. Weber, A. Wegrzynek, S. C. Wenzel, J. P. Wessels, J. Wiechula, J. Wikne, G. Wilk, J. Wilkinson, G. A. Willems, E. Willsher, B. Windelband, M. Winn, W. E. Witt, J. R. Wright, Y. Wu, R. Xu, S. Yalcin, Y. Yamaguchi, K. Yamakawa, S. Yang, S. Yano, Z. Yin, H. Yokoyama, I.-K. Yoo, J. H. Yoon, S. Yuan, A. Yuncu, V. Yurchenko, V. Zaccolo, A. Zaman, C. Zampolli, H. J. C. Zanoli, N. Zardoshti, A. Zarochentsev, P. Závada, N. Zaviyalov, H. Zbroszczyk, M. Zhalov, S. Zhang, X. Zhang, Z. Zhang, V. Zherebchevskii, Y. Zhi, D. Zhou, Y. Zhou, Z. Zhou, J. Zhu, Y. Zhu, A. Zichichi, G. Zinovjev, N. Zurlo

**Affiliations:** 1 Variable Energy Cyclotron Centre, Homi Bhabha National Institute, Kolkata, India; 2 Nuclear Physics Institute of the Czech Academy of Sciences, Řež, Czech Republic; 3 Institut für Informatik, Fachbereich Informatik und Mathematik, Johann-Wolfgang-Goethe Universität Frankfurt, Frankfurt am Main, Germany; 4 Division of Particle Physics, Department of Physics, Lund University, Lund, Sweden; 5 Physics Department, Panjab University, Chandigarh, India; 6 European Organization for Nuclear Research (CERN), Geneva, Switzerland; 7 Dipartimento DISAT del Politecnico and Sezione INFN, Turin, Italy; 8 Centro Fermi – Museo Storico della Fisica e Centro Studi e Ricerche “Enrico Fermi”, Rome, Italy; 9 INFN, Sezione di Bologna, Bologna, Italy; 10 Department of Physics, Aligarh Muslim University, Aligarh, India; 11 Korea Institute of Science and Technology Information, Daejeon, Republic of Korea; 12 Indonesian Institute of Sciences, Jakarta, Indonesia; 13 NRC «Kurchatov» Institute – ITEP, Moscow, Russia; 14 Research Division and ExtreMe Matter Institute EMMI, GSI Helmholtzzentrum für Schwerionenforschung, Darmstadt, Germany; 15 Fudan University, Shanghai, China; 16 Universidade Estadual de Campinas (UNICAMP), Campinas, Brazil; 17 National Research Centre Kurchatov Institute, Moscow, Russia; 18 INFN, Sezione di Torino, Turin, Italy; 19 Central China Normal University, Wuhan, China; 20 Instituto de Fsica, Universidad Nacional Autónoma de México, Mexico City, Mexico; 21 COMSATS University Islamabad, Islamabad, Pakistan; 22 Dipartimento di Fisica e Astronomia dell’Università and Sezione INFN, Bologna, Italy; 23 University of Houston, Houston, TX USA; 24 Bogolyubov Institute for Theoretical Physics, National Academy of Sciences of Ukraine, Kiev, Ukraine; 25 Department of Physics and Technology, University of Bergen, Bergen, Norway; 26 Institut für Kernphysik, Johann Wolfgang Goethe-Universität Frankfurt, Frankfurt am Main, Germany; 27 St. Petersburg State University, St. Petersburg, Russia; 28 Horia Hulubei National Institute of Physics and Nuclear Engineering, Bucharest, Romania; 29 Westfälische Wilhelms – Universität Münster, Institut für Kernphysik, Münster, Germany; 30 Physikalisches Institut, Ruprecht-Karls-Universität Heidelberg, Heidelberg, Germany; 31 Creighton University, Omaha, NB USA; 32 Rudjer Bošković Institute, Zagreb, Croatia; 33 INFN, Sezione di Padova, Padova, Italy; 34 Lawrence Berkeley National Laboratory, Berkeley, CA USA; 35 SUBATECH, IMT Atlantique, Université de Nantes, CNRS-IN2P3, Nantes, France; 36 Department of Physics, University of Oslo, Oslo, Norway; 37 Laboratoire de Physique des 2 Infinis, Irène Joliot-Curie, Orsay, France; 38 INFN, Sezione di Catania, Catania, Italy; 39 Gangneung-Wonju National University, Gangneung, Republic of Korea; 40 Physics Department, University of Jammu, Jammu, India; 41 Départment de Physique Nucléaire (DPhN), Université Paris-Saclay Centre d’Etudes de Saclay (CEA), IRFU, Saclay, France; 42 Helmholtz-Institut für Strahlen- und Kernphysik, Rheinische Friedrich-Wilhelms-Universität Bonn, Bonn, Germany; 43 Physik Department, Technische Universität München, Munich, Germany; 44 Bose Institute, Department of Physics and Centre for Astroparticle Physics and Space Science (CAPSS), Kolkata, India; 45 Dipartimento di Fisica e Astronomia dell’Università degli Studi di Catania and Sezione INFN, Catania, Italy; 46 Dipartimento di Fisica dell’Università degli studi di Torino and Sezione INFN, Turin, Italy; 47 Wigner Research Centre for Physics, Budapest, Hungary; 48 Nuclear Physics Group, STFC Daresbury Laboratory, Daresbury, UK; 49 Université Clermont Auvergne, CNRS/IN2P3, LPC, Clermont-Ferrand, France; 50 University of Liverpool, Liverpool, UK; 51 Dipartimento di Fisica e Astronomia dell’Università degli Studi di Padova and Sezione INFN, Padua, Italy; 52 Wayne State University, Detroit, MI USA; 53 Joint Institute for Nuclear Research (JINR), Dubna, Russia; 54 Indian Institute of Technology Bombay (IIT), Mumbai, India; 55 Sección Fsica, Departamento de Ciencias, Pontificia Universidad Católica del Perú, Lima, Peru; 56 Niels Bohr Institute, University of Copenhagen, Copenhagen, Denmark; 57 Yale University, New Haven, CT USA; 58 Institute for Subatomic Physics, Utrecht University/Nikhef, Utrecht, Netherlands; 59 Inha University, Incheon, Republic of Korea; 60 Université de Strasbourg, CNRS, IPHC UMR 7178 Strasbourg, France; 61 NRNU Moscow Engineering Physics Institute, Moscow, Russia; 62 Petersburg Nuclear Physics Institute, Gatchina, Russia; 63 University of Tennessee, Knoxville, TN USA; 64 Institute of Space Science (ISS), Bucharest, Romania; 65 Gauhati University, Department of Physics, Guwahati, India; 66 INFN, Laboratori Nazionali di Frascati, Frascati, Italy; 67 Faculty of Nuclear Sciences and Physical Engineering, Czech Technical University in Prague, Prague, Czech Republic; 68 The University of Texas at Austin, Austin, TX USA; 69 Università degli Studi di Pavia, Pavia, Italy; 70 Oak Ridge National Laboratory, Oak Ridge, TN USA; 71 Dipartimento di Fisica dell’Università degli studi di Cagliari and Sezione INFN, Cagliari, Italy; 72 Faculty of Science, P.J. Šafárik University, Košice, Slovakia; 73 Università di Brescia, Brescia, Italy; 74 Universidade de São Paulo (USP), São Paulo, Brazil; 75 Dipartimento Interateneo di Fisica ‘M. Merlin’, Università degli studi di Bari Aldo Moro and Sezione INFN, Bari, Italy; 76 Politecnico di Bari, Bari, Italy; 77 Russian Federal Nuclear Center (VNIIEF), Sarov, Russia; 78 Stefan Meyer Institut für Subatomare Physik (SMI), Vienna, Austria; 79 iThemba LABS, National Research Foundation, Somerset West, South Africa; 80 University of the Witwatersrand, Johannesburg, South Africa; 81 The Henryk Niewodniczanski Institute of Nuclear Physics, Polish Academy of Sciences, Krakow, Poland; 82 Nikhef, National Institute for Subatomic Physics, Amsterdam, Netherlands; 83 Universidad Autónoma de Sinaloa, Culiacán, Mexico; 84 High Energy Physics Group, Universidad Autónoma de Puebla, Puebla, Mexico; 85 Dipartimento di Fisica dell’Università degli studi di Trieste and Sezione INFN, Trieste, Italy; 86 INFN, Sezione di Cagliari, Cagliari, Italy; 87 Institute of Nuclear Physics, Homi Bhabha National Institute, Kolkata, India; 88 Université de Lyon, Université Lyon 1, CNRS/IN2P3, IPN-Lyon, Lyon, France; 89 University of Tsukuba, Tsukuba, Japan; 90 University of Cape Town, Cape Town, South Africa; 91 INFN, Sezione di Bari, Bari, Italy; 92 Laboratoire de Physique Subatomique et de Cosmologie, Université Grenoble-Alpes, CNRS-IN2P3, Grenoble, France; 93 INFN, Sezione di Trieste, Trieste, Italy; 94 Dipartimento di Scienze e Innovazione Tecnologica dell’Università del Piemonte Orientale and INFN Sezione di Torino, Alessandria, Italy; 95 Universidade Federal do ABC, Santo Andre, Brazil; 96 Instituto de Ciencias Nucleares, Universidad Nacional Autónoma de México, Mexico City, Mexico; 97 Warsaw University of Technology, Warsaw, Poland; 98 National Institute of Science Education and Research, Homi Bhabha National Institute, Jatni, India; 99 Dipartimento di Fisica ‘E.R. Caianiello’ dell’Università and Gruppo Collegato INFN, Salerno, Italy; 100 Frankfurt Institute for Advanced Studies, Johann Wolfgang Goethe-Universität Frankfurt, Frankfurt am Main, Germany; 101 Indian Institute of Technology Indore, Indore, India; 102 National Centre for Nuclear Research, Warsaw, Poland; 103 Centro de Aplicaciones Tecnológicas y Desarrollo Nuclear (CEADEN), Havana, Cuba; 104 Department of Physics, University of California, Berkeley, CA USA; 105 Institute for Nuclear Research, Academy of Sciences, Moscow, Russia; 106 Physics Department, Faculty of Science, University of Zagreb, Zagreb, Croatia; 107 School of Physics and Astronomy, University of Birmingham, Birmingham, UK; 108 NRC Kurchatov Institute IHEP, Protvino, Russia; 109 Department of Physics, School of Science, National and Kapodistrian University of Athens, Athens, Greece; 110 Chicago State University, Chicago, IL USA; 111 National Nuclear Research Center, Baku, Azerbaijan; 112 Instituto de Física, Universidade Federal do Rio Grande do Sul (UFRGS), Porto Alegre, Brazil; 113 Faculty of Electrical Engineering, Mechanical Engineering and Naval Architecture, University of Split, Split, Croatia; 114 A.I. Alikhanyan National Science Laboratory (Yerevan Physics Institute) Foundation, Yerevan, Armenia; 115 University of Tokyo, Tokyo, Japan; 116 Nagasaki Institute of Applied Science, Nagasaki, Japan; 117 Faculty of Engineering and Science, Western Norway University of Applied Sciences, Bergen, Norway; 118 Centro de Investigación y de Estudios Avanzados (CINVESTAV), Mexico City, Mexico; 119 Ohio State University, Columbus, OH USA; 120 Technical University of Košice, Košice, Slovakia; 121 Institute of Experimental Physics, Slovak Academy of Sciences, Košice, Slovakia; 122 KTO Karatay University, Konya, Turkey; 123 Zentrum für Technologietransfer und Telekommunikation (ZTT), Hochschule Worms, Worms, Germany; 124 Yonsei University, Seoul, Republic of Korea; 125 University of Jyväskylä, Jyväskylä, Finland; 126 Jeonbuk National University, Jeonju, Republic of Korea; 127 Department of Physics, Pusan National University, Pusan, Republic of Korea; 128 Department of Physics, Sejong University, Seoul, Republic of Korea; 129 California Polytechnic State University, San Luis Obispo, CA USA; 130 Suranaree University of Technology, Nakhon Ratchasima, Thailand; 131 University of South-Eastern Norway, Tonsberg, Norway; 132 China Institute of Atomic Energy, Beijing, China; 133 Dipartimento di Scienze MIFT, Università di Messina, Messina, Italy; 134 Institute of Physics of the Czech Academy of Sciences, Prague, Czech Republic; 135 Università degli Studi di Foggia, Foggia, Italy; 136 INFN, Sezione di Roma, Rome, Italy; 137 Dipartimento di Fisica dell’Università ‘La Sapienza’ and Sezione INFN, Rome, Italy; 138 Faculty of Mathematics, Physics and Informatics, Comenius University Bratislava, Bratislava, Slovakia; 139 Budker Institute for Nuclear Physics, Novosibirsk, Russia; 140 Physics Department, University of Rajasthan, Jaipur, India; 141 Helsinki Institute of Physics (HIP), Helsinki, Finland; 142 Institute of Physics, Homi Bhabha National Institute, Bhubaneswar, India; 143 Physikalisches Institut, Eberhard-Karls-Universität Tübingen, Tübingen, Germany; 144 Hiroshima University, Hiroshima, Japan; 145 Nara Women’s University (NWU), Nara, Japan; 146 Centre de Calcul de l’IN2P3, Lyon, France; 147 University of Science and Technology of China, Hefei, China; 148 Present Address: Italian National Agency for New Technologies, Energy and Sustainable Economic Development (ENEA), Bologna, Italy; 149 Present Address: Dipartimento DET, Politecnico di Torino, Turin, Italy; 150 Present Address: D.V. Skobeltsyn Institute of Nuclear Physics, M.V. Lomonosov Moscow State University, Moscow, Russia; 151 Present Address: Department of Applied Physics, Aligarh Muslim University, Aligarh, India; 152 Present Address: Institute of Theoretical Physics, University of Wrocław, Wrocław, Poland

**Keywords:** Experimental nuclear physics, Experimental particle physics

## Abstract

One of the key challenges for nuclear physics today is to understand from first principles the effective interaction between hadrons with different quark content. First successes have been achieved using techniques that solve the dynamics of quarks and gluons on discrete space-time lattices^1,2^. Experimentally, the dynamics of the strong interaction have been studied by scattering hadrons off each other. Such scattering experiments are difficult or impossible for unstable hadrons^3–6^ and so high-quality measurements exist only for hadrons containing up and down quarks^7^. Here we demonstrate that measuring correlations in the momentum space between hadron pairs^8–12^ produced in ultrarelativistic proton–proton collisions at the CERN Large Hadron Collider (LHC) provides a precise method with which to obtain the missing information on the interaction dynamics between any pair of unstable hadrons. Specifically, we discuss the case of the interaction of baryons containing strange quarks (hyperons). We demonstrate how, using precision measurements of proton–omega baryon correlations, the effect of the strong interaction for this hadron–hadron pair can be studied with precision similar to, and compared with, predictions from lattice calculations^13,14^. The large number of hyperons identified in proton–proton collisions at the LHC, together with accurate modelling^15^ of the small (approximately one femtometre) inter-particle distance and exact predictions for the correlation functions, enables a detailed determination of the short-range part of the nucleon-hyperon interaction.

## Abstract (web summary)

Correlations in momentum space between hadrons created by ultrarelativistic proton–proton collisions at the CERN Large Hadron Collider provide insights into the strong interaction, particularly the short-range dynamics of hyperons—baryons that contain strange quarks.

Baryons are composite objects formed by three valence quarks bound together by means of the strong interaction mediated through the emission and absorption of gluons. Between baryons, the strong interaction leads to a residual force and the most common example is the effective strong force among nucleons (*N*)—baryons composed of up (*u*) and down (*d*) quarks: proton (*p*) = *uud* and neutron (*n*) = *ddu*. This force is responsible for the existence of a neutron–proton bound state, the deuteron, and manifests itself in scattering experiments^7^ and through the existence of atomic nuclei. So far, our understanding of the nucleon–nucleon strong interaction relies heavily on effective theories^16^, where the degrees of freedom are nucleons. These effective theories are constrained by scattering measurements and are successfully used in the description of nuclear properties^17,18^.

The fundamental theory of the strong interaction is quantum chromodynamics (QCD), in which quarks and gluons are the degrees of freedom. One of the current challenges in nuclear physics is to calculate the strong interaction among hadrons starting from first principles. Perturbative techniques are used to calculate strong-interaction phenomena in high-energy collisions with a level of precision of a few per cent^19^. For baryon–baryon interactions at low energy such techniques cannot be employed; however, numerical solutions on a finite space-time lattice have been used to calculate scattering parameters among nucleons and the properties of light nuclei^1,2^. Such approaches are still limited: they do not yet reproduce the properties of the deuteron^20^ and do not predict physical values for the masses of light hadrons^21^.

Baryons containing strange (*s*) quarks, exclusively or combined with *u* and *d* quarks, are called hyperons (*Y*) and are denoted by uppercase Greek letters: *Λ* = *uds*, *Σ*^0^ = *uds*, *Ξ*^−^ = *dss*, *Ω*^−^ = *sss*. Experimentally, little is known about *Y*–*N* and *Y*–*Y* interactions, but recently, major steps forward in their understanding have been made using lattice QCD approaches^13,14,22^. The predictions available for hyperons are characterized by smaller uncertainties because the lattice calculation becomes more stable for quarks with larger mass, such as the *s* quark. In particular, robust results are obtained for interactions involving the heaviest hyperons, such as *Ξ* and *Ω*, and precise measurements of the *p*–*Ξ*^−^ and *p*–*Ω*^−^ interactions are instrumental in validating these calculations. From an experimental point of view, the existence of nuclei in which a nucleon is replaced by a hyperon (hypernuclei) demonstrates the presence of an attractive strong *Λ*–*N* interaction^23^ and indicates the possibility of binding a *Ξ*^−^ to a nucleus^24,25^. A direct and more precise measurement of the *Y*–*N* interaction requires scattering experiments, which are particularly challenging to perform because hyperons are short-lived and travel only a few centimetres before decaying. Previous experiments with *Λ* and *Σ* hyperons on proton targets^3–5^ delivered results that were two orders of magnitude less precise than those for nucleons, and such experiments with *Ξ* (ref. ^6^) and *Ω* beams are even more challenging. The measurement of the *Y*–*N* and *Y*–*Y* interactions has further important implications for the possible formation of a *Y*–*N* or *Y*–*Y* bound state. Although numerous theoretical predictions exist^13,26–30^, so far no clear evidence for any such bound states has been found, despite many experimental searches^31–35^.

Additionally, a precise knowledge of the *Y*–*N* and *Y*–*Y* interactions has important consequences for the physics of neutron stars. Indeed, the structure of the innermost core of neutron stars is still completely unknown and hyperons could appear in such environments depending on the *Y*–*N* and *Y*–*Y* interactions^36^. Real progress in this area calls for new experimental methods.

Studies of the *Y*–*N* interaction via correlations have been pioneered by the HADES collaboration^37^. Recently, the ALICE Collaboration has demonstrated that *p*–*p* and *p*–Pb collisions at the LHC are best suited to study the *N*–*N* and several *Y*–*N*, *Y*–*Y* interactions precisely^8–12^. Indeed, the collision energy and rate available at the LHC opens the phase space for an abundant production of any strange hadron^38^, and the capabilities of the ALICE detector for particle identification and the momentum resolution—with values below 1% for transverse momentum *p*_T_ < 1 GeV/*c*—facilitate the investigation of correlations in momentum space. These correlations reflect the properties of the interaction and hence can be used to test theoretical predictions by solving the Schrödinger equation for proton–hyperon collisions^39^. A fundamental advantage of *p*–*p* and *p*–Pb collisions at LHC energies is the fact that all hadrons originate from very small space-time volumes, with typical inter-hadron distances of about 1 fm. These small distances are linked through the uncertainty principle to a large range of the relative momentum (up to 200 MeV/*c*) for the baryon pair and enable us to test short-range interactions. Additionally, detailed modelling of a common source for all produced baryons^15^ allow us to determine accurately the source parameters.

Similar studies were carried out in ultrarelativistic Au–Au collisions at a centre-of-mass energy of 200 GeV per nucleon pair by the STAR collaboration for *Λ*–*Λ*^40,41^ and *p*–*Ω*^−^^42^ interactions. This collision system leads to comparatively large particle emitting sources of 3–5 fm. The resulting relative momentum range is below 40 MeV/*c*, implying reduced sensitivity to interactions at distances shorter than 1 fm.

In this work, we present a precision study of the most exotic among the proton–hyperon interactions, obtained via the *p*–*Ω*^−^ correlation function in *p*–*p* collisions at a centre-of-mass energy $\sqrt{s}=13\,{\rm{TeV}}$ at the LHC. The comparison of the measured correlation function with first-principle calculations^13^ and with a new precision measurement of the *p*–*Ξ*^−^ correlation in the same collision system provides the first observation of the effect of the strong interaction for the *p*–*Ω*^−^ pair. The implications of the measured correlations for a possible *p*–*Ω*^−^ bound state are also discussed. These experimental results challenge the interpretation of the data in terms of lattice QCD as the precision of the data improves.

Our measurement opens a new chapter for experimental methods in hadron physics with the potential to pin down the strong interaction for all known proton–hyperon pairs.

## Analysis of the correlation function

**Fig. 1:**
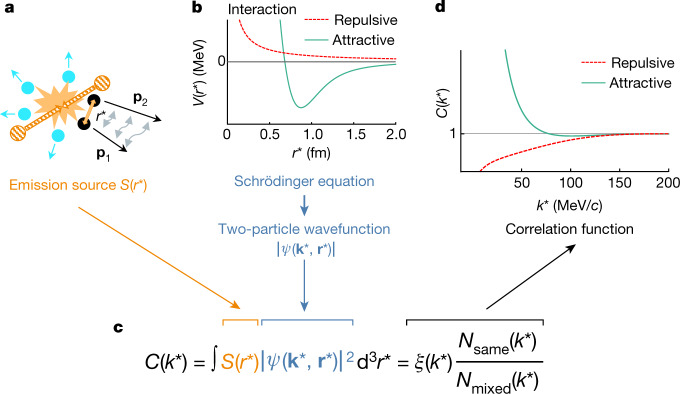
Schematic representation of the correlation method. **a**, A collision of two protons generates a particle source *S*(r*) from which a hadron–hadron pair with momenta **p**_1_ and **p**_2_ emerges at a relative distance r* and can undergo a final-state interaction before being detected. Consequently, the relative momentum k* is either reduced or increased via an attractive or a repulsive interaction, respectively. **b**, Example of attractive (green) and repulsive (dotted red) interaction potentials, *V*(r*), between two hadrons, as a function of their relative distance. Given a certain potential, a non-relativistic Schrödinger equation is used to obtain the corresponding two-particle wavefunction, *ψ*(k*, r*). **c**, The equation of the calculated (second term) and measured (third term) correlation function *C*(k*), where *N*_same_(k*) and *N*_mixed_(k*) represent the k* distributions of hadron–hadron pairs produced in the same and in different collisions, respectively, and *ξ*(k*) denotes the corrections for experimental effects. **d**, Sketch of the resulting shape of *C*(k*). The value of the correlation function is proportional to the interaction strength. It is above unity for an attractive (green) potential, and between zero and unity for a repulsive (dotted red) potential.

Figure 1 shows a schematic representation of the correlation method used in this analysis. The correlation function can be expressed theoretically^43,44^ as *C*(k*) = ∫d^3^r*S(r*) × |*ψ*(k*, r*)|^2^, where k* and r* are the relative momentum and relative distance of the pair of interest. *S*(r*) is the distribution of the distance r* = |r*| at which particles are emitted (defining the source size), *ψ*(k*, r*) represents the wavefunction of the relative motion for the pair of interest and k* = |k*| is the reduced relative momentum of the pair (${k}^{\ast }=|{{\bf{p}}}_{2}^{\ast }-{{\bf{p}}}_{1}^{\ast }|/2$). Given an interaction potential between two hadrons as a function of their relative distance, a non-relativistic Schrödinger equation can be used^39^ to obtain the corresponding wavefunction and hence also predict the expected correlation function. The choice of a non-relativistic Schrödinger equation is motivated by the fact that the typical relative momenta relevant for the strong final-state interaction have a maximal value of 200 MeV/*c*. Experimentally, this correlation function is computed as *C*(k*) = *ξ*(k*)[*N*_same_(k*)/*N*_mixed_(k*)], where *ξ*(k*) denotes the corrections for experimental effects, *N*_same_(k*) is the number of pairs with a given k* obtained by combining particles produced in the same collision (event), which constitute a sample of correlated pairs, and *N*_mixed_(k*) is the number of uncorrelated pairs with the same k*, obtained by combining particles produced in different collisions (the so-called mixed-event technique). Figure 1d shows how an attractive or repulsive interaction is mapped into the correlation function. For an attractive interaction the magnitude of the correlation function will be above unity for small values of k*, whereas for a repulsive interaction it will be between zero and unity. In the former case, the presence of a bound state would create a depletion of the correlation function with a depth increasing with increasing binding energy.

Correlations can occur in nature from quantum mechanical interference, resonances, conservation laws or final-state interactions. Here, it is the final-state interactions that contribute predominantly at low relative momentum; in this work we focus on the strong and Coulomb interactions in pairs composed of a proton and either a *Ξ*^−^ or a *Ω*^−^ hyperon.

**Fig. 2:**
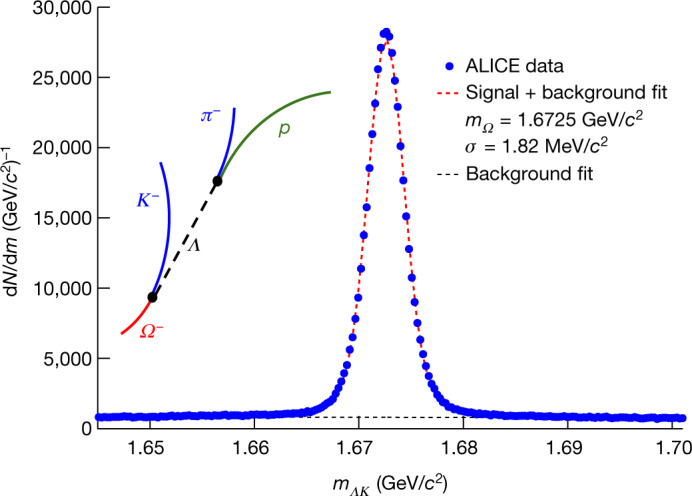
Reconstruction of the *Ω*^−^ and ${\bar{{\boldsymbol{\Omega }}}}^{+}$ signals. Sketch of the weak decay of *Ω*^−^ into a *Λ* and a *Κ*^−^, and measured invariant mass distribution (blue points) of *ΛΚ*^−^ and $\bar{\varLambda }{K}^{+}$ combinations. The dotted red line represents the fit to the data including signal and background, and the black dotted line the background alone. The contamination from misidentification is ≤5%.

Protons do not decay and can hence be directly identified within the ALICE detector, but *Ξ*^−^ and *Ω*^−^ baryons are detected through their weak decays, *Ξ*^−^ → *Λ* + *π*^−^ and *Ω*^−^ → *Λ* + *Κ*^−^. The identification and momentum measurement of protons, *Ξ*^−^, *Ω*^−^ and their respective antiparticles are described in Methods. Figure 2 shows a sketch of the *Ω*^−^ decay and the invariant mass distribution of the *ΛΚ*^−^ and $\bar{\varLambda }{K}^{+}$ pairs. The clear peak corresponding to the rare *Ω*^−^ and ${\bar{\varOmega }}^{+}$ baryons demonstrates the excellent identification capability, which is the key ingredient for this measurement. The contamination from misidentification is ≤5%. For the *Ξ*^−^ (${\bar{\varXi }}^{+}$) baryon the misidentification amounts to 8%^11^.

Once the *p*, *Ω*^−^ and *Ξ*^−^ candidates and charge conjugates are selected and their 3-momenta measured, the correlation functions can be built. Since we assume that the same interaction governs baryon–baryon and antibaryon–antibaryon pairs^8^, we consider in the following the direct sum (⊕) of particles and antiparticles ($p{\textstyle \mbox{--}}{\varXi }^{-}\oplus \bar{p}{\textstyle \mbox{--}}{\bar{\varXi }}^{+}\equiv p{\textstyle \mbox{--}}{\varXi }^{-}$ and $p{\textstyle \mbox{--}}{\varOmega }^{-}\oplus \bar{p}{\textstyle \mbox{--}}{\bar{\varOmega }}^{+}\equiv p{\textstyle \mbox{--}}{\varOmega }^{-}$). The determination of the correction *ξ*(k*) and the evaluation of the systematic uncertainties are described in Methods.

## Comparison of the *p*–*Ξ*^−^ and *p*–*Ω*^−^ interactions

**Fig. 3:**
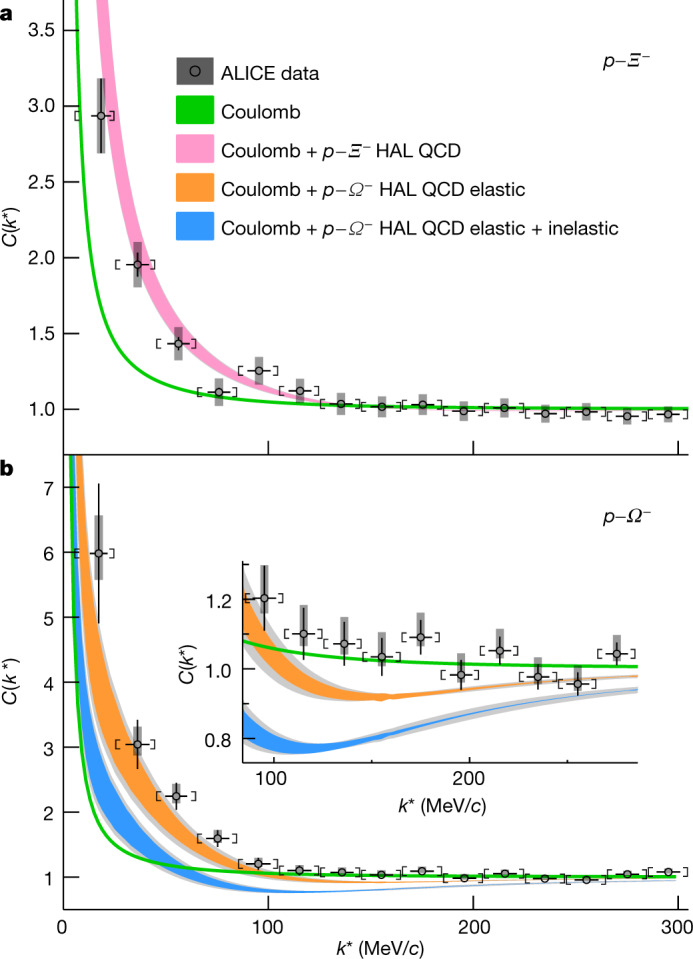
Experimental *p*–*Ξ*^−^ and *p*–*Ω*^−^ correlation functions. **a**, **b**, Measured *p*–*Ξ*^−^ (**a**) and *p*–*Ω*^−^ (**b**) correlation functions in high multiplicity *p*–*p* collisions at $\sqrt{s}=13\,{\rm{TeV}}$ . The experimental data are shown as black symbols. The black vertical bars and the grey boxes represent the statistical and systematic uncertainties. The square brackets show the bin width and the horizontal black lines represent the statistical uncertainty in the determination of the mean k* for each bin. The measurements are compared with theoretical predictions, shown as coloured bands, that assume either Coulomb or Coulomb + strong HAL QCD interactions. For the *p*–*Ω*^−^ system the orange band represents the prediction considering only the elastic contributions and the blue band represents the prediction considering both elastic and inelastic contributions. The width of the curves including HAL QCD predictions represents the uncertainty associated with the calculation (see Methods section ‘Corrections of the correlation function’ for details) and the grey shaded band represents, in addition, the uncertainties associated with the determination of the source radius. The width of the Coulomb curves represents only the uncertainty associated with the source radius. The considered radius values are 1.02 ± 0.05 fm for *p*–*Ξ*^−^ and 0.95 ± 0.06 fm for *p*–*Ω*^−^ pairs, respectively. The inset in **b** shows an expanded view of the *p*–*Ω*^−^ correlation function for *C*(k*) close to unity. For more details see text.

The obtained correlation functions are shown in Fig. 3a, b for the *p*–*Ξ*^−^ and *p*–*Ω*^−^ pairs, respectively, along with the statistical and systematic uncertainties. The fact that both correlations are well above unity implies the presence of an attractive interaction for both systems. For opposite-charge pairs, as considered here, the Coulomb interaction is attractive and its effect on the correlation function is illustrated by the green curves in both panels of Fig. 3. These curves have been obtained by solving the Schrödinger equation for *p*–*Ξ*^−^ and *p*–*Ω*^−^ pairs using the Correlation Analysis Tool using the Schrödinger equation (CATS) equation solver^39^, considering only the Coulomb interaction and assuming that the shape of the source follows a Gaussian distribution with a width equal to 1.02 ± 0.05 fm for the *p*–*Ξ*^−^ system and to 0.95 ± 0.06 fm for the *p*–*Ω*^−^ system, respectively. The source-size values have been determined via an independent analysis of *p*–*p* correlations^15^, where modifications of the source distribution due to strong decays of short-lived resonances are taken into account, and the source size is determined as a function of the transverse mass *m*_T_ of the pair, as described in Methods. The average *m*_T_ of the *p*–*Ξ*^−^ and *p*–*Ω*^−^ pairs are 1.9 GeV/*c* and 2.2 GeV/*c*, respectively. The difference in size between the source of the *p*–*Ξ*^−^ and *p*–*Ω*^−^ pairs might reflect the contribution of collective effects such as (an)isotropic flow. The width of the green curves in Fig. 3 reflects the quoted uncertainty of the measured source radius. The correlations obtained, accounting only for the Coulomb interaction, considerably underestimate the strength of both measured correlations. This implies, in both cases, that an attractive interaction exists and exceeds the strength of the Coulomb interaction.

**Fig. 4:**
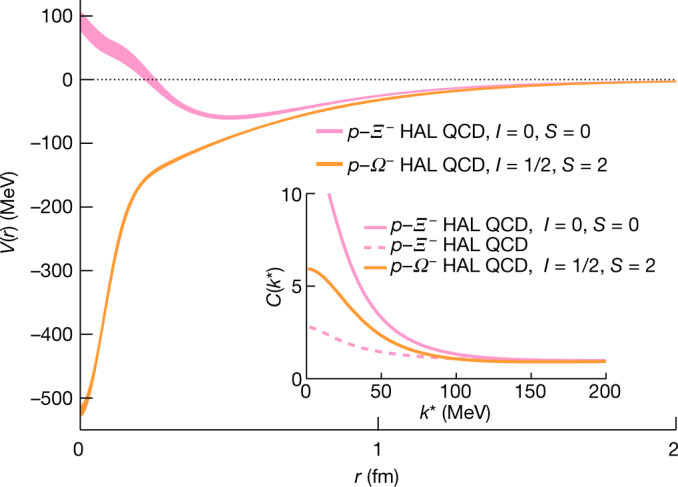
Potentials for the *p*–*Ξ*^−^ and *p*–*Ω*^−^ interactions. *p*–*Ξ*^−^ (pink) and *p*–*Ω*^−^ (orange) interaction potentials as a function of the pair distance predicted by the HAL QCD collaboration^13,14^. Only the most attractive component, isospin *I* = 0 and spin *S* = 0, is shown for *p*–*Ξ*^−^. For the *p*–*Ω*^−^ interaction the *I* = 1/2 and spin *S* = 2 component is shown. The widths of the curves correspond to the uncertainties (see Methods section ‘Corrections of the correlation function’ for details) associated with the calculations. The inset shows the correlation functions obtained using the HAL QCD strong interaction potentials for: (i) the channel *p*–*Ξ*^−^ with isospin *I* = 0 and spin *S* = 0, (ii) the channel *p*–*Ξ*^−^ including all allowed spin and isospin combinations (dashed pink), and (iii) the channel *p*–*Ω*^−^ with isospin *I* = 1/2 and spin *S* = 2. For details see text.

To discuss the comparison of the experimental data with the predictions from lattice QCD, it is useful to first focus on the distinct characteristics of the *p*–*Ξ*^−^ and *p*–*Ω*^−^ interactions. Figure 4 shows the radial shapes obtained for the strong-interaction potentials calculated from first principles by the HAL QCD (Hadrons to Atomic nuclei from Lattice QCD) collaboration for the *p*–*Ξ*^−^ (ref. ^14^) and the *p*–*Ω*^−^ systems^13^, see Methods for details. Only the most attractive (isospin *I* = 0 and spin *S* = 0) of the four components^14^ of the *p*–*Ξ*^−^ interaction and the isospin *I* = 1/2 and spin *S* = 2 component of the *p*–*Ω*^−^ interaction are shown. Aside from an attractive component, we see that the interaction contains also a repulsive core starting at very small distances, below 0.2 fm. For the *p*–*Ω*^−^ system no repulsive core is visible and the interaction is purely attractive. This very attractive interaction can accommodate a *p*–*Ω*^−^ bound state, with a binding energy of about 2.5 MeV, considering the Coulomb and strong forces^13^. The *p*–*Ξ*^−^ and *p*–*Ω*^−^ interaction potentials look very similar to each other above a distance of 1 fm. This behaviour is not observed in phenomenological models that engage the exchange of heavy mesons and predict a quicker fall off of the potentials^45^.

The inset of Fig. 4 shows the correlation functions obtained using the HAL QCD strong interaction potentials for: (i) the channel *p*–*Ξ*^−^ with isospin *I* = 0 and spin *S* = 0, (ii) the channel *p*–*Ξ*^−^ including all allowed spin and isospin combinations, and (iii) the channel *p*–*Ω*^−^ with isospin *I* = 1/2 and spin *S* = 2. The correlation functions are computed using the experimental values for the *p*–*Ξ*^−^ and *p*–*Ω*^−^ source-size. Despite the fact that the strong *p*–*Ω*^−^ potential is more attractive than the *p*–*Ξ*^−^ *I* = 0 and *S* = 0 potential, the resulting correlation function is lower. This is due to the presence of the bound state in the *p*–*Ω*^−^ case^46^. If we consider all four isospin and spin components of the *p*–*Ξ*^−^ interaction^11^ the prediction for the global *p*–*Ξ*^−^ correlation function is lower than that for *p*–*Ω*^−^. Experimentally, as shown in Fig. 3, the less attractive strong *p*–*Ξ*^−^ interaction translates into a correlation function that reaches values of 3 in comparison with the much higher values of up to 6 that are visible for the *p*–*Ω*^−^ correlation. The theoretical predictions shown in Fig. 3 also include the effect of the Coulomb interaction.

Regarding the *p*–*Ξ*^−^ interaction, it should be considered that strangeness-rearrangement processes can occur, such as *pΞ*^−^ → *ΛΛ*, *ΣΣ*, *ΛΣ*. This means that the inverse processes (for example, *ΛΛ* → *pΞ*^−^) can also occur and modify the *p*–*Ξ*^−^ correlation function. These contributions are accounted for within lattice calculations by exploiting the well known quark symmetries^14^ and are found to be very small. Moreover, the ALICE collaboration measured the *Λ*–*Λ* correlation in *p*–*p* and *p*–Pb collisions^10^ and good agreement with the shallow interaction predicted by the HAL QCD collaboration was found.

The resulting prediction for the correlation function, obtained by solving the Schrödinger equation for the single *p*–*Ξ*^−^ channel including the HAL QCD strong and Coulomb interactions, is shown in Fig. 3a. The first measurement of the *p*–*Ξ*^−^ interaction using *p*–Pb collisions^11^ showed a qualitative agreement to lattice QCD predictions. The improved precision of the data in the current analysis of *p*–*p* collisions is also in agreement with calculations that include both the HAL QCD and Coulomb interactions.

## Detailed study of the *p*–*Ω*^−^ correlation

Concerning the *p*–*Ω*^−^ interaction, strangeness-rearrangement processes can also occur^47^, such as *pΩ*^−^ → *ΞΛ*, *ΞΣ*. Such processes might affect the *p*–*Ω*^−^ interaction in a different way depending on the relative orientation of the total spin and angular momentum of the pair. Since the proton has *J*_*p*_ = 1/2 and the *Ω* has *J*_*Ω*_ = 3/2 and the orbital angular momentum *L* can be neglected for correlation studies that imply low relative momentum, the total angular momentum *J* equals the total spin *S* and can take on values of *J* = 2 or *J* = 1. The *J* = 2 state cannot couple to the strangeness-rearrangement processes discussed above, except through D-wave processes, which are strongly suppressed. For the *J* = 1 state only two limiting cases can be discussed in the absence of measurements of the *pΩ*^−^ → *ΞΛ*, *ΞΣ* cross-sections.

The first case assumes that the effect of the inelastic channels is negligible for both configurations and that the radial behaviour of the interaction is driven by elastic processes, following the lattice QCD potential (see Fig. 4), for both the *J* = 2 and *J* = 1 channels. This results in a prediction, shown by the orange curve in Fig. 3b, that is close to the data in the low k* region. The second limiting case assumes, following a previous prescription^47^, that the *J* = 1 configuration is completely dominated by strangeness-rearrangement processes. The obtained correlation function is shown by the blue curve Fig. 3b. This curve clearly deviates from the data. Both theoretical calculations also include the effect of the Coulomb interaction and they predict the existence of a *p*–*Ω*^−^ bound state with a binding energy of 2.5 MeV, which causes a depletion in the correlation function in the k* region between 100 and 300 MeV/*c*, because pairs that form a bound state are lost to the correlation yield. The inset of Fig. 3 shows that in this k* region the data are consistent with unity and do not follow either of the two theoretical predictions.

At the moment, the lattice QCD predictions underestimate the data, but additional measurements are necessary to draw a firm conclusion on the existence of the bound state. Measurements of *Λ*–*Ξ*^−^ and *Σ*^0^–*Ξ*^−^ correlations will verify experimentally the strength of possible non-elastic contributions. Measurements of the *p*–*Ω*^−^ correlation function in collision systems with slightly larger size (for example, *p*–Pb collisions at the LHC)^11^ will clarify the possible presence of a depletion in *C*(k*). Indeed, the appearance of a depletion in the correlation function depends on the interplay between the average intra-particle distance (source size) and the scattering length associated with the *p*–*Ω*^−^ interaction^47^.

## Summary

We have shown that the hyperon–proton interaction can be studied in unprecedented detail in *p*–*p* collisions at $\sqrt{s}=13\,{\rm{TeV}}$ at the LHC. We have demonstrated, in particular, that even the as-yet-unknown *p*–*Ω*^−^ interaction can be investigated with excellent precision. The comparison of the measured correlation functions shows that the *p*–*Ω*^−^ signal is up to a factor two larger than the *p*–*Ξ*^−^ signal. This reflects the large difference in the strong-attractive interaction predicted by the first-principle calculations by the HAL QCD collaboration. The correlation functions predicted by HAL QCD are in agreement with the measurements for the *p*–*Ξ*^−^ interaction. For the *p*–*Ω*^−^ interaction, the inelastic channels are not yet accounted for quantitatively within the lattice QCD calculations. Additionally, the depletion in the correlation function that is visible in the calculations around k* = 150 MeV/*c*, owing to the presence of a *p*–*Ω*^−^ bound state, is not observed in the measured correlation. To draw quantitative conclusions concerning the existence of a *p*–*Ω*^−^ bound state, we plan a direct measurement of the *Λ*–*Ξ*^−^ and *Σ*^0^−*Ξ*^−^ correlations and a study of the *p*–*Ω*^−^ correlation in *p*–Pb collisions in the near future. Indeed, with the upgraded ALICE apparatus^48^ and the increased data sample size expected from the high luminosity phase of the LHC Run 3 and Run 4^49^, the missing interactions involving hyperons will be measured in *p*–*p* and *p*–Pb collisions and this should enable us to answer the question about the existence of a new baryon–baryon bound state. Since this method can be extended to almost any hadron–hadron pair, an unexpected avenue for high-precision tests of the strong interaction at the LHC has been opened.

## Methods

### Event selection

Events were recorded from inelastic *p*–*p* collisions by ALICE^50,51^ at the LHC. A trigger that requires the total signal amplitude measured in the V0 detector^52^ to exceed a certain threshold was used to select high-multiplicity (HM) events. The V0 detector comprises two plastic scintillator arrays placed on both sides of the interaction point at pseudorapidities 2.8 < *η* < 5.1 and −3.7 < *η* < −1.7. The pseudorapidity is defined as *η* = −ln[tan(*θ*/2)], where *θ* is the polar angle of the particle with respect to the proton beam axis.

At $\sqrt{s}=13\,{\rm{TeV}}$, in the HM events, 30 charged particles in the range |*η*| < 0.5 are produced on average. This *η* range corresponds to the region within 26 degrees of the transverse plane that is perpendicular to the beam axis. The HM events are rare, constituting 0.17% of the *p*–*p* collisions that produce at least one charged particle in the pseudorapidity range |*η*| < 1.0. It was shown^38^ that HM events contain an enhanced yield of hyperons, which facilitates this analysis. The yield of *Ω*^−^ in HM events is at least a factor 5 larger, on average, compared with that in total inelastic collisions^53^. A total of 1 × 10^9^ HM events were analysed. Additional details on the HM event selection can be found in a previous work^12^.

### Particle tracking and identification

For the identification and momentum measurement of charged particles, the Inner Tracking System (ITS)^54^, Time Projection Chamber (TPC)^55^, and Time-Of-Flight (TOF)^56^ detectors of ALICE are used. All three detectors are located inside a solenoid magnetic field (0.5 T) leading to a bending of the trajectories of charged particles. The measurement of the curvature is used to reconstruct the particle momenta. Typical transverse momentum (*p*_T_) resolutions for protons, pions and kaons vary from about 2% for tracks with *p*_T_ = 10 GeV/*c* to below 1% for *p*_T_ < 1 GeV/*c*. The particle identity is determined by the energy lost per unit of track length inside the TPC detector and, in some cases, by the particle velocity measured in the TOF detector. Additional experimental details are discussed in a previous work^51^.

Protons are selected within a transverse momentum range of 0.5 < *p*_T_ < 4.05 GeV/*c*. They are identified requiring TPC information for candidate tracks with momentum *p* < 0.75 GeV/*c*, whereas TPC and TOF information are both required for candidates with *p* > 0.75 GeV/*c*. An incorrect identification of primary protons occurs in 1% of the cases, as evaluated by Monte Carlo simulations.

Direct tracking and identification is not possible for *Ξ*^−^ and *Ω*^−^ hyperons and their antiparticles, because they are unstable and decay as a result of the weak interaction within a few centimetres after their production. The mean decay distances (evaluated as *c* × *τ*, where *τ* is the particle lifetime) of ${\varXi }^{-}({\bar{\varXi }}^{+})\to \varLambda (\bar{\varLambda })+{{\pi }}^{-}({{\pi }}^{+})$ and ${\varOmega }^{-}({\bar{\varOmega }}^{+})\to \varLambda (\bar{\varLambda })+{K}^{-}({K}^{+})$ are 4.9 and 2.5 cm, respectively^57^. Both decays are followed by a second decay of the unstable $\varLambda (\bar{\varLambda })$ hyperon, $\varLambda (\bar{\varLambda })\to p(\bar{p})+{{\pi }}^{-}({{\pi }}^{+})$, with an average decay path of 7.9 cm (ref. ^57^). Consequently, pions (*π*^±^), kaons (*Κ*^±^) and protons have to be detected and then combined to search for ${\varXi }^{-}({\bar{\varXi }}^{+})$ and ${\varOmega }^{-}({\bar{\varOmega }}^{+})$ candidates. Those secondary particles are identified by the TPC information in the case of the reconstruction of ${\varXi }^{-}({\bar{\varXi }}^{+})$, and in the case of ${\varOmega }^{-}({\bar{\varOmega }}^{+})$ it is additionally required that the secondary protons and kaons are identified in the TOF detector. To measure the ${\varXi }^{-}({\bar{\varXi }}^{+})$ and ${\varOmega }^{-}({\bar{\varOmega }}^{+})$  hyperons, the two successive weak decays need to be reconstructed. The reconstruction procedure is very similar for both hyperons and is described in detail previously^58^. Topological selections are performed to reduce the combinatorial background, evaluated via a fit to the invariant mass distribution.

### Determination of the source size

The widths of the Gaussian distributions constituting *S*(r*), and defining the source size, are calculated on the basis of the results of the analysis of the *p*–*p* correlation function in *p*–*p* collisions at $\sqrt{s}=13\,{\rm{TeV}}$ by the ALICE collaboration^15^. Assuming a common source for all baryons, its size was studied as a function of the transverse mass of the baryon–baryon pair, ${m}_{{\rm{T}}}={({k}_{{\rm{T}}}^{2}+{m}^{2})}^{1/2}$, where *m* is the average mass and *k*_T_ = |**p**_T,1_ + **p**_T,2_|/2 is the transverse momentum of the pair. The source size decreases with increasing mass, which could reflect the collective evolution of the system. The average transverse mass ⟨*m*_T_⟩ for the *p*–*Ξ*^−^ and *p*–*Ω*^−^ pairs differ and are equal to 1.9 GeV/*c* and 2.2 GeV/*c*, respectively. To determine the source sizes for these values, the measurement from *p*–*p* correlations (shown in figure 5 of ref. ^15^) is parameterized as ${r}_{{\rm{core}}}=a{m}_{{\rm{T}}}^{b}+c$, where *r*_core_ denotes the width of the Gaussian distribution defining the source before taking into account the effect produced by short lived resonances.

In *p*–*p* collisions at $\sqrt{s}=13\,{\rm{TeV}}$, *Ξ*^−^ and *Ω*^−^ baryons are produced mostly as primary particles, but about 2/3 of the protons originate from the decay of short-lived resonances with a lifetime of a few fm per *c*. As a result, the effective source size of both *p*–*Ξ*^−^ and *p*–*Ω*^−^ is modified. This effect is taken into account by folding the Gaussian source with an exponential distribution following the method outlined previously^15^. The resulting source distribution can be characterized by an effective Gaussian source radius equal to 1.02 ± 0.05 fm for *p*–*Ξ*^−^ pairs and to 0.95 ± 0.06 fm for *p*–*Ω*^−^ pairs. The quoted uncertainties correspond to variations of the parametrization of the *p*–*p* results according to their systematic and statistical uncertainties.

### Corrections of the correlation function

The correction factor *ξ*(k*) accounts for the normalization of the k* distribution of pairs from mixed-events, for effects produced by finite momentum resolution and for the influence of residual correlations.

The mixed-event distribution, *N*_mixed_(k*), has to be scaled down, because the number of pairs available from mixed events is much higher than the number of pairs produced in the same collision used in *N*_same_(k*). The normalization parameter ${\mathscr{N}}$ is chosen such that the mean value of the correlation function equals to unity in a region of k* values where the effect of final-state interactions are negligible, 500 < k* < 800 MeV/*c*.

The finite experimental momentum resolution modifies the measured correlation functions at most by 8% at low k*. A correction for this effect is applied. Resolution effects due to the merging of tracks that are very close to each other were evaluated and found to be negligible.

The two measured correlation functions are dominated by the contribution of the interaction between *p*–*Ξ*^−^ and *p*–*Ω*^−^ pairs. Nevertheless, other contributions also influence the measured correlation function. They originate either from incorrectly identified particles or from particles stemming from other weak decays (such as protons from *Λ* → *p* + *π*^−^ decays) combined with primary particles. Because weak decays occur typically some centimetres away from the collision vertex, there is no final-state interaction between their decay products and the primary particles of interest. Hence, the resulting correlation function either will be completely flat or will carry the residual signature of the interaction between the particles before the decay. A method to determine the exact shape and relative yields of the residual correlations has been previously developed^8,59^, and it is used in this analysis. Such contributions are subtracted from the measured *p*–*Ξ*^−^ and *p*–*Ω*^−^ correlations to obtain the genuine correlation functions. The residual correlation stemming from misidentification is evaluated experimentally^11^ and its contribution is also subtracted from the measured correlation function.

The systematic uncertainties associated with the genuine correlation function arise from the following sources: (i) the selection of the proton, ${\varXi }^{-}({\bar{\varXi }}^{+})$ and ${\varOmega }^{-}({\bar{\varOmega }}^{+})$, (ii) the normalization of the mixed-event distributions, (iii) uncertainties on the residual contributions, and (iv) uncertainties due to the finite momentum resolution. To evaluate the associated systematic uncertainties: (i) all single-particle and topological selection criteria are varied with respect to their default values and the analysis is repeated for 50 different random combinations of such selection criteria so that the maximum change introduced in the number of *p*–*Ξ*^−^ and *p*–*Ω*^−^ pairs is 25% and the changes in the purity of protons, ${\varXi }^{-}({\bar{\varXi }}^{+})$ and ${\varOmega }^{-}({\bar{\varOmega }}^{+})$ are kept below 3%; (ii) the k*-normalization range of the mixed-events is varied, and a linear function of k* is also used for an alternative normalization which results in an asymmetric uncertainty; (iii) the shape of the residual correlations and its relative contribution are altered; and (iv) the momentum resolution and the used correction method are changed. The total systematic uncertainties associated with the genuine correlation function are maximal at low k*, reaching a value of 9% and 8% for *p*–*Ξ*^−^ and *p*–*Ω*^−^, respectively.

### HAL QCD potentials

Results from calculations by the HAL QCD Collaboration for the *p*–*Ξ*^−^^14^ and *p*–*Ω*^−^^13^ interactions are shown in Figs. 3, 4. Such interactions were studied via (2 + 1)-flavor lattice QCD simulations with nearly physical quark masses (*m*_*π*_ = 146 MeV/*c*^2^).

In Fig. 4, the *p*–*Ξ*^−^ and *p*–*Ω*^−^ potentials are shown for calculations with *t*/*a* = 12, with *t* the Euclidean time and *a* the lattice spacing of the calculations. The HAL QCD Collaboration provided 23 and 20 sets of parameters for the description of the shape of the *p*–*Ξ*^−^ and *p*–*Ω*^−^ potentials, respectively. Such parametrizations result from applying the jackknife method, which takes into account the statistical uncertainty of the calculations. The width of the curves in Fig. 4 corresponds to the maximum variations observed in the potential shape by using the different sets of parameters.

To obtain the correlation functions shown in Fig. 3 we consider the calculations with *t*/*a* = 12, both for *p*–*Ξ*^−^ and *p*–*Ω*^−^. The statistical uncertainty of the calculations is evaluated using the jackknife variations, and a systematic uncertainty is added in quadrature evaluated by considering calculations with *t*/*a* = 11 and *t*/*a* = 13.

## Online content

Any methods, additional references, Nature Research reporting summaries, source data, extended data, supplementary information, acknowledgements, peer review information; details of author contributions and competing interests; and statements of data and code availability are available at 10.1038/s41586-020-3001-6.

## Data Availability

All data shown in histograms and plots are publicly available on the HEPdata repository (https://hepdata.net).

## Code availability

The source code used in this study is publicly available under the names AliPhysics (https://github.com/alisw/AliPhysics) and AliRoot (https://github.com/alisw/AliROOT). Further information can be provided by the authors upon reasonable request.
