# Publisher Correction: Unveiling the strong interaction among hadrons at the LHC

**DOI:** 10.1038/s41586-020-03142-2

**Published:** 2021-01-15

**Authors:** ALICE Collaboration, S. Acharya, D. Adamová, A. Adler, J. Adolfsson, M. M. Aggarwal, G. Aglieri Rinella, M. Agnello, N. Agrawal, Z. Ahammed, S. Ahmad, S. U. Ahn, Z. Akbar, A. Akindinov, M. Al-Turany, S. N. Alam, D. S. D. Albuquerque, D. Aleksandrov, B. Alessandro, H. M. Alfanda, R. Alfaro Molina, B. Ali, Y. Ali, A. Alici, N. Alizadehvandchali, A. Alkin, J. Alme, T. Alt, L. Altenkamper, I. Altsybeev, M. N. Anaam, C. Andrei, D. Andreou, A. Andronic, M. Angeletti, V. Anguelov, C. Anson, T. Antičić, F. Antinori, P. Antonioli, N. Apadula, L. Aphecetche, H. Appelshäuser, S. Arcelli, R. Arnaldi, M. Arratia, I. C. Arsene, M. Arslandok, A. Augustinus, R. Averbeck, S. Aziz, M. D. Azmi, A. Badalà, Y. W. Baek, S. Bagnasco, X. Bai, R. Bailhache, R. Bala, A. Balbino, A. Baldisseri, M. Ball, S. Balouza, D. Banerjee, R. Barbera, L. Barioglio, G. G. Barnaföldi, L. S. Barnby, V. Barret, P. Bartalini, C. Bartels, K. Barth, E. Bartsch, F. Baruffaldi, N. Bastid, S. Basu, G. Batigne, B. Batyunya, D. Bauri, J. L. Bazo Alba, I. G. Bearden, C. Beattie, C. Bedda, N. K. Behera, I. Belikov, A. D. C. Bell Hechavarria, F. Bellini, R. Bellwied, V. Belyaev, G. Bencedi, S. Beole, A. Bercuci, Y. Berdnikov, D. Berenyi, R. A. Bertens, D. Berzano, M. G. Besoiu, L. Betev, A. Bhasin, I. R. Bhat, M. A. Bhat, H. Bhatt, B. Bhattacharjee, A. Bianchi, L. Bianchi, N. Bianchi, J. Bielčík, J. Bielčíková, A. Bilandzic, G. Biro, R. Biswas, S. Biswas, J. T. Blair, D. Blau, C. Blume, G. Boca, F. Bock, A. Bogdanov, S. Boi, J. Bok, L. Boldizsár, A. Bolozdynya, M. Bombara, G. Bonomi, H. Borel, A. Borissov, H. Bossi, E. Botta, L. Bratrud, P. Braun-Munzinger, M. Bregant, M. Broz, E. Bruna, G. E. Bruno, M. D. Buckland, D. Budnikov, H. Buesching, S. Bufalino, O. Bugnon, P. Buhler, P. Buncic, Z. Buthelezi, J. B. Butt, S. A. Bysiak, D. Caffarri, A. Caliva, E. Calvo Villar, J. M. M. Camacho, R. S. Camacho, P. Camerini, F. D. M. Canedo, A. A. Capon, F. Carnesecchi, R. Caron, J. Castillo Castellanos, A. J. Castro, E. A. R. Casula, F. Catalano, C. Ceballos Sanchez, P. Chakraborty, S. Chandra, W. Chang, S. Chapeland, M. Chartier, S. Chattopadhyay, S. Chattopadhyay, A. Chauvin, C. Cheshkov, B. Cheynis, V. Chibante Barroso, D. D. Chinellato, S. Cho, P. Chochula, T. Chowdhury, P. Christakoglou, C. H. Christensen, P. Christiansen, T. Chujo, C. Cicalo, L. Cifarelli, L. D. Cilladi, F. Cindolo, M. R. Ciupek, G. Clai, J. Cleymans, F. Colamaria, D. Colella, A. Collu, M. Colocci, M. Concas, G. Conesa Balbastre, Z. Conesa del Valle, G. Contin, J. G. Contreras, T. M. Cormier, Y. Corrales Morales, P. Cortese, M. R. Cosentino, F. Costa, S. Costanza, P. Crochet, E. Cuautle, P. Cui, L. Cunqueiro, D. Dabrowski, T. Dahms, A. Dainese, F. P. A. Damas, M. C. Danisch, A. Danu, D. Das, I. Das, P. Das, P. Das, S. Das, A. Dash, S. Dash, S. De, A. De Caro, G. de Cataldo, J. de Cuveland, A. De Falco, D. De Gruttola, N. De Marco, S. De Pasquale, S. Deb, H. F. Degenhardt, K. R. Deja, A. Deloff, S. Delsanto, W. Deng, P. Dhankher, D. Di Bari, A. Di Mauro, R. A. Diaz, T. Dietel, P. Dillenseger, Y. Ding, R. Divià, D. U. Dixit, Ø. Djuvsland, U. Dmitrieva, A. Dobrin, B. Dönigus, O. Dordic, A. K. Dubey, A. Dubla, S. Dudi, M. Dukhishyam, P. Dupieux, R. J. Ehlers, V. N. Eikeland, D. Elia, B. Erazmus, F. Erhardt, A. Erokhin, M. R. Ersdal, B. Espagnon, G. Eulisse, D. Evans, S. Evdokimov, L. Fabbietti, M. Faggin, J. Faivre, F. Fan, A. Fantoni, M. Fasel, P. Fecchio, A. Feliciello, G. Feofilov, A. Fernández Téllez, A. Ferrero, A. Ferretti, A. Festanti, V. J. G. Feuillard, J. Figiel, S. Filchagin, D. Finogeev, F. M. Fionda, G. Fiorenza, F. Flor, A. N. Flores, S. Foertsch, P. Foka, S. Fokin, E. Fragiacomo, U. Frankenfeld, U. Fuchs, C. Furget, A. Furs, M. Fusco Girard, J. J. Gaardhøje, M. Gagliardi, A. M. Gago, A. Gal, C. D. Galvan, P. Ganoti, C. Garabatos, J. R. A. Garcia, E. Garcia-Solis, K. Garg, C. Gargiulo, A. Garibli, K. Garner, P. Gasik, E. F. Gauger, M. B. Gay Ducati, M. Germain, J. Ghosh, P. Ghosh, S. K. Ghosh, M. Giacalone, P. Gianotti, P. Giubellino, P. Giubilato, A. M. C. Glaenzer, P. Glässel, A. Gomez Ramirez, V. Gonzalez, L. H. González-Trueba, S. Gorbunov, L. Görlich, A. Goswami, S. Gotovac, V. Grabski, L. K. Graczykowski, K. L. Graham, L. Greiner, A. Grelli, C. Grigoras, V. Grigoriev, A. Grigoryan, S. Grigoryan, O. S. Groettvik, F. Grosa, J. F. Grosse-Oetringhaus, R. Grosso, R. Guernane, M. Guittiere, K. Gulbrandsen, T. Gunji, A. Gupta, R. Gupta, I. B. Guzman, R. Haake, M. K. Habib, C. Hadjidakis, H. Hamagaki, G. Hamar, M. Hamid, R. Hannigan, M. R. Haque, A. Harlenderova, J. W. Harris, A. Harton, J. A. Hasenbichler, H. Hassan, Q. U. Hassan, D. Hatzifotiadou, P. Hauer, L. B. Havener, S. Hayashi, S. T. Heckel, E. Hellbär, H. Helstrup, A. Herghelegiu, T. Herman, E. G. Hernandez, G. Herrera Corral, F. Herrmann, K. F. Hetland, H. Hillemanns, C. Hills, B. Hippolyte, B. Hohlweger, J. Honermann, D. Horak, A. Hornung, S. Hornung, R. Hosokawa, P. Hristov, C. Huang, C. Hughes, P. Huhn, T. J. Humanic, H. Hushnud, L. A. Husova, N. Hussain, S. A. Hussain, D. Hutter, J. P. Iddon, R. Ilkaev, H. Ilyas, M. Inaba, G. M. Innocenti, M. Ippolitov, A. Isakov, M. S. Islam, M. Ivanov, V. Ivanov, V. Izucheev, B. Jacak, N. Jacazio, P. M. Jacobs, S. Jadlovska, J. Jadlovsky, S. Jaelani, C. Jahnke, M. J. Jakubowska, M. A. Janik, T. Janson, M. Jercic, O. Jevons, M. Jin, F. Jonas, P. G. Jones, J. Jung, M. Jung, A. Jusko, P. Kalinak, A. Kalweit, V. Kaplin, S. Kar, A. Karasu Uysal, D. Karatovic, O. Karavichev, T. Karavicheva, P. Karczmarczyk, E. Karpechev, A. Kazantsev, U. Kebschull, R. Keidel, M. Keil, B. Ketzer, Z. Khabanova, A. M. Khan, S. Khan, A. Khanzadeev, Y. Kharlov, A. Khatun, A. Khuntia, B. Kileng, B. Kim, B. Kim, D. Kim, D. J. Kim, E. J. Kim, H. Kim, J. Kim, J. S. Kim, J. Kim, J. Kim, J. Kim, M. Kim, S. Kim, T. Kim, T. Kim, S. Kirsch, I. Kisel, S. Kiselev, A. Kisiel, J. L. Klay, C. Klein, J. Klein, S. Klein, C. Klein-Bösing, M. Kleiner, A. Kluge, M. L. Knichel, A. G. Knospe, C. Kobdaj, M. K. Köhler, T. Kollegger, A. Kondratyev, N. Kondratyeva, E. Kondratyuk, J. Konig, S. A. Konigstorfer, P. J. Konopka, G. Kornakov, L. Koska, O. Kovalenko, V. Kovalenko, M. Kowalski, I. Králik, A. Kravčáková, L. Kreis, M. Krivda, F. Krizek, K. Krizkova Gajdosova, M. Krüger, E. Kryshen, M. Krzewicki, A. M. Kubera, V. Kučera, C. Kuhn, P. G. Kuijer, L. Kumar, S. Kundu, P. Kurashvili, A. Kurepin, A. B. Kurepin, A. Kuryakin, S. Kushpil, J. Kvapil, M. J. Kweon, J. Y. Kwon, Y. Kwon, S. L. La Pointe, P. La Rocca, Y. S. Lai, M. Lamanna, R. Langoy, K. Lapidus, A. Lardeux, P. Larionov, E. Laudi, R. Lavicka, T. Lazareva, R. Lea, L. Leardini, J. Lee, S. Lee, S. Lehner, J. Lehrbach, R. C. Lemmon, I. León Monzón, E. D. Lesser, M. Lettrich, P. Lévai, X. Li, X. L. Li, J. Lien, R. Lietava, B. Lim, V. Lindenstruth, A. Lindner, C. Lippmann, M. A. Lisa, A. Liu, J. Liu, S. Liu, W. J. Llope, I. M. Lofnes, V. Loginov, C. Loizides, P. Loncar, J. A. Lopez, X. Lopez, E. López Torres, J. R. Luhder, M. Lunardon, G. Luparello, Y. G. Ma, A. Maevskaya, M. Mager, S. M. Mahmood, T. Mahmoud, A. Maire, R. D. Majka, M. Malaev, Q. W. Malik, L. Malinina, D. Mal’Kevich, P. Malzacher, G. Mandaglio, V. Manko, F. Manso, V. Manzari, Y. Mao, M. Marchisone, J. Mareš, G. V. Margagliotti, A. Margotti, A. Marín, C. Markert, M. Marquard, C. D. Martin, N. A. Martin, P. Martinengo, J. L. Martinez, M. I. Martínez, G. Martínez García, S. Masciocchi, M. Masera, A. Masoni, L. Massacrier, E. Masson, A. Mastroserio, A. M. Mathis, O. Matonoha, P. F. T. Matuoka, A. Matyja, C. Mayer, F. Mazzaschi, M. Mazzilli, M. A. Mazzoni, A. F. Mechler, F. Meddi, Y. Melikyan, A. Menchaca-Rocha, C. Mengke, E. Meninno, A. S. Menon, M. Meres, S. Mhlanga, Y. Miake, L. Micheletti, L. C. Migliorin, D. L. Mihaylov, K. Mikhaylov, A. N. Mishra, D. Miśkowiec, A. Modak, N. Mohammadi, A. P. Mohanty, B. Mohanty, M. Mohisin Khan, Z. Moravcova, C. Mordasini, D. A. Moreira De Godoy, L. A. P. Moreno, I. Morozov, A. Morsch, T. Mrnjavac, V. Muccifora, E. Mudnic, D. Mühlheim, S. Muhuri, J. D. Mulligan, A. Mulliri, M. G. Munhoz, R. H. Munzer, H. Murakami, S. Murray, L. Musa, J. Musinsky, C. J. Myers, J. W. Myrcha, B. Naik, R. Nair, B. K. Nandi, R. Nania, E. Nappi, M. U. Naru, A. F. Nassirpour, C. Nattrass, R. Nayak, T. K. Nayak, S. Nazarenko, A. Neagu, R. A. Negrao De Oliveira, L. Nellen, S. V. Nesbo, G. Neskovic, D. Nesterov, L. T. Neumann, B. S. Nielsen, S. Nikolaev, S. Nikulin, V. Nikulin, F. Noferini, P. Nomokonov, J. Norman, N. Novitzky, P. Nowakowski, A. Nyanin, J. Nystrand, M. Ogino, A. Ohlson, J. Oleniacz, A. C. Oliveira Da Silva, M. H. Oliver, C. Oppedisano, A. Ortiz Velasquez, A. Oskarsson, J. Otwinowski, K. Oyama, Y. Pachmayer, V. Pacik, S. Padhan, D. Pagano, G. Paić, J. Pan, S. Panebianco, P. Pareek, J. Park, J. E. Parkkila, S. Parmar, S. P. Pathak, B. Paul, J. Pazzini, H. Pei, T. Peitzmann, X. Peng, L. G. Pereira, H. Pereira Da Costa, D. Peresunko, G. M. Perez, S. Perrin, Y. Pestov, V. Petráček, M. Petrovici, R. P. Pezzi, S. Piano, M. Pikna, P. Pillot, O. Pinazza, L. Pinsky, C. Pinto, S. Pisano, D. Pistone, M. Płoskoń, M. Planinic, F. Pliquett, M. G. Poghosyan, B. Polichtchouk, N. Poljak, A. Pop, S. Porteboeuf-Houssais, V. Pozdniakov, S. K. Prasad, R. Preghenella, F. Prino, C. A. Pruneau, I. Pshenichnov, M. Puccio, J. Putschke, S. Qiu, L. Quaglia, R. E. Quishpe, S. Ragoni, S. Raha, S. Rajput, J. Rak, A. Rakotozafindrabe, L. Ramello, F. Rami, S. A. R. Ramirez, R. Raniwala, S. Raniwala, S. S. Räsänen, R. Rath, V. Ratza, I. Ravasenga, K. F. Read, A. R. Redelbach, K. Redlich, A. Rehman, P. Reichelt, F. Reidt, X. Ren, R. Renfordt, Z. Rescakova, K. Reygers, A. Riabov, V. Riabov, T. Richert, M. Richter, P. Riedler, W. Riegler, F. Riggi, C. Ristea, S. P. Rode, M. Rodríguez Cahuantzi, K. Røed, R. Rogalev, E. Rogochaya, D. Rohr, D. Röhrich, P. F. Rojas, P. S. Rokita, F. Ronchetti, A. Rosano, E. D. Rosas, K. Roslon, A. Rossi, A. Rotondi, A. Roy, P. Roy, O. V. Rueda, R. Rui, B. Rumyantsev, A. Rustamov, E. Ryabinkin, Y. Ryabov, A. Rybicki, H. Rytkonen, O. A. M. Saarimaki, R. Sadek, S. Sadhu, S. Sadovsky, K. Šafařík, S. K. Saha, B. Sahoo, P. Sahoo, R. Sahoo, S. Sahoo, P. K. Sahu, J. Saini, S. Sakai, S. Sambyal, V. Samsonov, D. Sarkar, N. Sarkar, P. Sarma, V. M. Sarti, M. H. P. Sas, E. Scapparone, J. Schambach, H. S. Scheid, C. Schiaua, R. Schicker, A. Schmah, C. Schmidt, H. R. Schmidt, M. O. Schmidt, M. Schmidt, N. V. Schmidt, A. R. Schmier, J. Schukraft, Y. Schutz, K. Schwarz, K. Schweda, G. Scioli, E. Scomparin, J. E. Seger, Y. Sekiguchi, D. Sekihata, I. Selyuzhenkov, S. Senyukov, D. Serebryakov, A. Sevcenco, A. Shabanov, A. Shabetai, R. Shahoyan, W. Shaikh, A. Shangaraev, A. Sharma, A. Sharma, H. Sharma, M. Sharma, N. Sharma, S. Sharma, O. Sheibani, K. Shigaki, M. Shimomura, S. Shirinkin, Q. Shou, Y. Sibiriak, S. Siddhanta, T. Siemiarczuk, D. Silvermyr, G. Simatovic, G. Simonetti, B. Singh, R. Singh, R. Singh, R. Singh, V. K. Singh, V. Singhal, T. Sinha, B. Sitar, M. Sitta, T. B. Skaali, M. Slupecki, N. Smirnov, R. J. M. Snellings, C. Soncco, J. Song, A. Songmoolnak, F. Soramel, S. Sorensen, I. Sputowska, J. Stachel, I. Stan, P. J. Steffanic, E. Stenlund, S. F. Stiefelmaier, D. Stocco, M. M. Storetvedt, L. D. Stritto, A. A. P. Suaide, T. Sugitate, C. Suire, M. Suleymanov, M. Suljic, R. Sultanov, M. Šumbera, V. Sumberia, S. Sumowidagdo, S. Swain, A. Szabo, I. Szarka, U. Tabassam, S. F. Taghavi, G. Taillepied, J. Takahashi, G. J. Tambave, S. Tang, M. Tarhini, M. G. Tarzila, A. Tauro, G. Tejeda Muñoz, A. Telesca, L. Terlizzi, C. Terrevoli, D. Thakur, S. Thakur, D. Thomas, F. Thoresen, R. Tieulent, A. Tikhonov, A. R. Timmins, A. Toia, N. Topilskaya, M. Toppi, F. Torales-Acosta, S. R. Torres, A. Trifiró, S. Tripathy, T. Tripathy, S. Trogolo, G. Trombetta, L. Tropp, V. Trubnikov, W. H. Trzaska, T. P. Trzcinski, B. A. Trzeciak, A. Tumkin, R. Turrisi, T. S. Tveter, K. Ullaland, E. N. Umaka, A. Uras, G. L. Usai, M. Vala, N. Valle, S. Vallero, N. van der Kolk, L. V. R. van Doremalen, M. van Leeuwen, P. Vande Vyvre, D. Varga, Z. Varga, M. Varga-Kofarago, A. Vargas, M. Vasileiou, A. Vasiliev, O. Vázquez Doce, V. Vechernin, E. Vercellin, S. Vergara Limón, L. Vermunt, R. Vernet, R. Vértesi, L. Vickovic, Z. Vilakazi, O. Villalobos Baillie, G. Vino, A. Vinogradov, T. Virgili, V. Vislavicius, A. Vodopyanov, B. Volkel, M. A. Völkl, K. Voloshin, S. A. Voloshin, G. Volpe, B. von Haller, I. Vorobyev, D. Voscek, J. Vrláková, B. Wagner, M. Weber, S. G. Weber, A. Wegrzynek, S. C. Wenzel, J. P. Wessels, J. Wiechula, J. Wikne, G. Wilk, J. Wilkinson, G. A. Willems, E. Willsher, B. Windelband, M. Winn, W. E. Witt, J. R. Wright, Y. Wu, R. Xu, S. Yalcin, Y. Yamaguchi, K. Yamakawa, S. Yang, S. Yano, Z. Yin, H. Yokoyama, I.-K. Yoo, J. H. Yoon, S. Yuan, A. Yuncu, V. Yurchenko, V. Zaccolo, A. Zaman, C. Zampolli, H. J. C. Zanoli, N. Zardoshti, A. Zarochentsev, P. Závada, N. Zaviyalov, H. Zbroszczyk, M. Zhalov, S. Zhang, X. Zhang, Z. Zhang, V. Zherebchevskii, Y. Zhi, D. Zhou, Y. Zhou, Z. Zhou, J. Zhu, Y. Zhu, A. Zichichi, G. Zinovjev, N. Zurlo

**Affiliations:** 1 Variable Energy Cyclotron Centre, Homi Bhabha National Institute, Kolkata, India; 2 Nuclear Physics Institute of the Czech Academy of Sciences, Řež, Czech Republic; 3 Institut für Informatik, Fachbereich Informatik und Mathematik, Johann-Wolfgang-Goethe Universität Frankfurt, Frankfurt am Main, Germany; 4 Division of Particle Physics, Department of Physics, Lund University, Lund, Sweden; 5 Physics Department, Panjab University, Chandigarh, India; 6 European Organization for Nuclear Research (CERN), Geneva, Switzerland; 7 Dipartimento DISAT del Politecnico and Sezione INFN, Turin, Italy; 8 Centro Fermi – Museo Storico della Fisica e Centro Studi e Ricerche “Enrico Fermi”, Rome, Italy; 9 INFN, Sezione di Bologna, Bologna, Italy; 10 Department of Physics, Aligarh Muslim University, Aligarh, India; 11 Korea Institute of Science and Technology Information, Daejeon, Republic of Korea; 12 Indonesian Institute of Sciences, Jakarta, Indonesia; 13 NRC «Kurchatov» Institute – ITEP, Moscow, Russia; 14 Research Division and ExtreMe Matter Institute EMMI, GSI Helmholtzzentrum für Schwerionenforschung, Darmstadt, Germany; 15 Fudan University, Shanghai, China; 16 Universidade Estadual de Campinas (UNICAMP), Campinas, Brazil; 17 National Research Centre Kurchatov Institute, Moscow, Russia; 18 INFN, Sezione di Torino, Turin, Italy; 19 Central China Normal University, Wuhan, China; 20 Instituto de Fsica, Universidad Nacional Autónoma de México, Mexico City, Mexico; 21 COMSATS University Islamabad, Islamabad, Pakistan; 22 Dipartimento di Fisica e Astronomia dell’Università and Sezione INFN, Bologna, Italy; 23 University of Houston, Houston, TX USA; 24 Bogolyubov Institute for Theoretical Physics, National Academy of Sciences of Ukraine, Kiev, Ukraine; 25 Department of Physics and Technology, University of Bergen, Bergen, Norway; 26 Institut für Kernphysik, Johann Wolfgang Goethe-Universität Frankfurt, Frankfurt am Main, Germany; 27 St. Petersburg State University, St. Petersburg, Russia; 28 Horia Hulubei National Institute of Physics and Nuclear Engineering, Bucharest, Romania; 29 Westfälische Wilhelms – Universität Münster, Institut für Kernphysik, Münster, Germany; 30 Physikalisches Institut, Ruprecht-Karls-Universität Heidelberg, Heidelberg, Germany; 31 Creighton University, Omaha, NB USA; 32 Rudjer Bošković Institute, Zagreb, Croatia; 33 INFN, Sezione di Padova, Padova, Italy; 34 Lawrence Berkeley National Laboratory, Berkeley, CA USA; 35 SUBATECH, IMT Atlantique, Université de Nantes, CNRS-IN2P3, Nantes, France; 36 Department of Physics, University of Oslo, Oslo, Norway; 37 Laboratoire de Physique des 2 Infinis, Irène Joliot-Curie, Orsay, France; 38 INFN, Sezione di Catania, Catania, Italy; 39 Gangneung-Wonju National University, Gangneung, Republic of Korea; 40 Physics Department, University of Jammu, Jammu, India; 41 Départment de Physique Nucléaire (DPhN), Université Paris-Saclay Centre d’Etudes de Saclay (CEA), IRFU, Saclay, France; 42 Helmholtz-Institut für Strahlen- und Kernphysik, Rheinische Friedrich-Wilhelms-Universität Bonn, Bonn, Germany; 43 Physik Department, Technische Universität München, Munich, Germany; 44 Bose Institute, Department of Physics and Centre for Astroparticle Physics and Space Science (CAPSS), Kolkata, India; 45 Dipartimento di Fisica e Astronomia dell’Università degli Studi di Catania and Sezione INFN, Catania, Italy; 46 Dipartimento di Fisica dell’Università degli studi di Torino and Sezione INFN, Turin, Italy; 47 Wigner Research Centre for Physics, Budapest, Hungary; 48 Nuclear Physics Group, STFC Daresbury Laboratory, Daresbury, UK; 49 Université Clermont Auvergne, CNRS/IN2P3, LPC, Clermont-Ferrand, France; 50 University of Liverpool, Liverpool, UK; 51 Dipartimento di Fisica e Astronomia dell’Università degli Studi di Padova and Sezione INFN, Padua, Italy; 52 Wayne State University, Detroit, MI USA; 53 Joint Institute for Nuclear Research (JINR), Dubna, Russia; 54 Indian Institute of Technology Bombay (IIT), Mumbai, India; 55 Sección Fsica, Departamento de Ciencias, Pontificia Universidad Católica del Perú, Lima, Peru; 56 Niels Bohr Institute, University of Copenhagen, Copenhagen, Denmark; 57 Yale University, New Haven, CT USA; 58 Institute for Subatomic Physics, Utrecht University/Nikhef, Utrecht, Netherlands; 59 Inha University, Incheon, Republic of Korea; 60 Université de Strasbourg, CNRS, IPHC UMR 7178 Strasbourg, France; 61 NRNU Moscow Engineering Physics Institute, Moscow, Russia; 62 Petersburg Nuclear Physics Institute, Gatchina, Russia; 63 University of Tennessee, Knoxville, TN USA; 64 Institute of Space Science (ISS), Bucharest, Romania; 65 Gauhati University, Department of Physics, Guwahati, India; 66 INFN, Laboratori Nazionali di Frascati, Frascati, Italy; 67 Faculty of Nuclear Sciences and Physical Engineering, Czech Technical University in Prague, Prague, Czech Republic; 68 The University of Texas at Austin, Austin, TX USA; 69 Università degli Studi di Pavia, Pavia, Italy; 70 Oak Ridge National Laboratory, Oak Ridge, TN USA; 71 Dipartimento di Fisica dell’Università degli studi di Cagliari and Sezione INFN, Cagliari, Italy; 72 Faculty of Science, P.J. Šafárik University, Košice, Slovakia; 73 Università di Brescia, Brescia, Italy; 74 Universidade de São Paulo (USP), São Paulo, Brazil; 75 Dipartimento Interateneo di Fisica ‘M. Merlin’, Università degli studi di Bari Aldo Moro and Sezione INFN, Bari, Italy; 76 Politecnico di Bari, Bari, Italy; 77 Russian Federal Nuclear Center (VNIIEF), Sarov, Russia; 78 Stefan Meyer Institut für Subatomare Physik (SMI), Vienna, Austria; 79 iThemba LABS, National Research Foundation, Somerset West, South Africa; 80 University of the Witwatersrand, Johannesburg, South Africa; 81 The Henryk Niewodniczanski Institute of Nuclear Physics, Polish Academy of Sciences, Krakow, Poland; 82 Nikhef, National Institute for Subatomic Physics, Amsterdam, Netherlands; 83 Universidad Autónoma de Sinaloa, Culiacán, Mexico; 84 High Energy Physics Group, Universidad Autónoma de Puebla, Puebla, Mexico; 85 Dipartimento di Fisica dell’Università degli studi di Trieste and Sezione INFN, Trieste, Italy; 86 INFN, Sezione di Cagliari, Cagliari, Italy; 87 Institute of Nuclear Physics, Homi Bhabha National Institute, Kolkata, India; 88 Université de Lyon, Université Lyon 1, CNRS/IN2P3, IPN-Lyon, Lyon, France; 89 University of Tsukuba, Tsukuba, Japan; 90 University of Cape Town, Cape Town, South Africa; 91 INFN, Sezione di Bari, Bari, Italy; 92 Laboratoire de Physique Subatomique et de Cosmologie, Université Grenoble-Alpes, CNRS-IN2P3, Grenoble, France; 93 INFN, Sezione di Trieste, Trieste, Italy; 94 Dipartimento di Scienze e Innovazione Tecnologica dell’Università del Piemonte Orientale and INFN Sezione di Torino, Alessandria, Italy; 95 Universidade Federal do ABC, Santo Andre, Brazil; 96 Instituto de Ciencias Nucleares, Universidad Nacional Autónoma de México, Mexico City, Mexico; 97 Warsaw University of Technology, Warsaw, Poland; 98 National Institute of Science Education and Research, Homi Bhabha National Institute, Jatni, India; 99 Dipartimento di Fisica ‘E.R. Caianiello’ dell’Università and Gruppo Collegato INFN, Salerno, Italy; 100 Frankfurt Institute for Advanced Studies, Johann Wolfgang Goethe-Universität Frankfurt, Frankfurt am Main, Germany; 101 Indian Institute of Technology Indore, Indore, India; 102 National Centre for Nuclear Research, Warsaw, Poland; 103 Centro de Aplicaciones Tecnológicas y Desarrollo Nuclear (CEADEN), Havana, Cuba; 104 Department of Physics, University of California, Berkeley, CA USA; 105 Institute for Nuclear Research, Academy of Sciences, Moscow, Russia; 106 Physics Department, Faculty of Science, University of Zagreb, Zagreb, Croatia; 107 School of Physics and Astronomy, University of Birmingham, Birmingham, UK; 108 NRC Kurchatov Institute IHEP, Protvino, Russia; 109 Department of Physics, School of Science, National and Kapodistrian University of Athens, Athens, Greece; 110 Chicago State University, Chicago, IL USA; 111 National Nuclear Research Center, Baku, Azerbaijan; 112 Instituto de Física, Universidade Federal do Rio Grande do Sul (UFRGS), Porto Alegre, Brazil; 113 Faculty of Electrical Engineering, Mechanical Engineering and Naval Architecture, University of Split, Split, Croatia; 114 A.I. Alikhanyan National Science Laboratory (Yerevan Physics Institute) Foundation, Yerevan, Armenia; 115 University of Tokyo, Tokyo, Japan; 116 Nagasaki Institute of Applied Science, Nagasaki, Japan; 117 Faculty of Engineering and Science, Western Norway University of Applied Sciences, Bergen, Norway; 118 Centro de Investigación y de Estudios Avanzados (CINVESTAV), Mexico City, Mexico; 119 Ohio State University, Columbus, OH USA; 120 Technical University of Košice, Košice, Slovakia; 121 Institute of Experimental Physics, Slovak Academy of Sciences, Košice, Slovakia; 122 KTO Karatay University, Konya, Turkey; 123 Zentrum für Technologietransfer und Telekommunikation (ZTT), Hochschule Worms, Worms, Germany; 124 Yonsei University, Seoul, Republic of Korea; 125 University of Jyväskylä, Jyväskylä, Finland; 126 Jeonbuk National University, Jeonju, Republic of Korea; 127 Department of Physics, Pusan National University, Pusan, Republic of Korea; 128 Department of Physics, Sejong University, Seoul, Republic of Korea; 129 California Polytechnic State University, San Luis Obispo, CA USA; 130 Suranaree University of Technology, Nakhon Ratchasima, Thailand; 131 University of South-Eastern Norway, Tonsberg, Norway; 132 China Institute of Atomic Energy, Beijing, China; 133 Dipartimento di Scienze MIFT, Università di Messina, Messina, Italy; 134 Institute of Physics of the Czech Academy of Sciences, Prague, Czech Republic; 135 Università degli Studi di Foggia, Foggia, Italy; 136 INFN, Sezione di Roma, Rome, Italy; 137 Dipartimento di Fisica dell’Università ‘La Sapienza’ and Sezione INFN, Rome, Italy; 138 Faculty of Mathematics, Physics and Informatics, Comenius University Bratislava, Bratislava, Slovakia; 139 Budker Institute for Nuclear Physics, Novosibirsk, Russia; 140 Physics Department, University of Rajasthan, Jaipur, India; 141 Helsinki Institute of Physics (HIP), Helsinki, Finland; 142 Institute of Physics, Homi Bhabha National Institute, Bhubaneswar, India; 143 Physikalisches Institut, Eberhard-Karls-Universität Tübingen, Tübingen, Germany; 144 Hiroshima University, Hiroshima, Japan; 145 Nara Women’s University (NWU), Nara, Japan; 146 Centre de Calcul de l’IN2P3, Lyon, France; 147 University of Science and Technology of China, Hefei, China; 148 Present Address: Italian National Agency for New Technologies, Energy and Sustainable Economic Development (ENEA), Bologna, Italy; 149 Present Address: Dipartimento DET, Politecnico di Torino, Turin, Italy; 150 Present Address: D.V. Skobeltsyn Institute of Nuclear Physics, M.V. Lomonosov Moscow State University, Moscow, Russia; 151 Present Address: Department of Applied Physics, Aligarh Muslim University, Aligarh, India; 152 Present Address: Institute of Theoretical Physics, University of Wrocław, Wrocław, Poland

**Keywords:** Experimental nuclear physics, Experimental particle physics

Correction to: *Nature* 10.1038/s41586-020-3001-6Published online 09 December 2020

In Fig. 1c of this Article, owing to an error during the production process, the equation incorrectly began ‘*C*(k*, r*) = …’ instead of ‘*C*(k*) = …’. In addition, in affiliation 71 ‘Dipartimento di Fisica dell’Università degli studi di Bari Aldo Moro’ has been corrected to read ‘Dipartimento di Fisica dell’Università degli studi di Cagliari’. The original Article has been corrected online.

A list of authors and their affiliations appears online.

